# Novel Benzenesulfonate Scaffolds with a High Anticancer Activity and G2/M Cell Cycle Arrest

**DOI:** 10.3390/cancers13081790

**Published:** 2021-04-09

**Authors:** Katarzyna Malarz, Jacek Mularski, Michał Kuczak, Anna Mrozek-Wilczkiewicz, Robert Musiol

**Affiliations:** 1 A. Chełkowski Institute of Physics and Silesian Centre for Education and Interdisciplinary Research, University of Silesia in Katowice, 75 Pułku Piechoty 1a, 41-500 Chorzów, Poland; mkuczak@us.edu.pl (M.K.); anna.mrozek-wilczkiewicz@us.edu.pl (A.M.-W.); 2 Institute of Chemistry, University of Silesia in Katowice, 75 Pułku Piechoty 1a, 41-500 Chorzów, Poland; jacek.mularski@us.edu.pl

**Keywords:** styrylquinazoline, sulfonic derivatives, anticancer activity, cell cycle inhibition, G2/M phase, apoptosis, autophagy

## Abstract

**Simple Summary:**

Sulfonate derivatives have limited application in pharmacology. Only few examples of small-molecule alkylating agents used as DNA poisons are known. This is the first report presenting strong anticancer activity of aromatic sulfonates based on quinazolines. The screening revealed that compounds expressed good submicromolar activity exceeding imatinib against a panel of cancer cell lines, including leukemia, colon, pancreatic cancers and glioblastoma, and minimal effect on proliferation of non-cancer cells. This activity corresponds with strong cell cycle arrest and mitotic inhibition similar or higher than that of paclitaxel. Further investigation revealed a more multitargeted mechanism of action. This structure may be an effective, novel scaffold for drug design.

**Abstract:**

Sulfonates, unlike their derivatives, sulphonamides, have rarely been investigated for their anticancer activity. Unlike the well-known sulphonamides, esters are mainly used as convenient intermediates in a synthesis. Here, we present the first in-depth investigation of quinazoline sulfonates. A small series of derivatives were synthesized and tested for their anticancer activity. Based on their structural similarity, these compounds resemble tyrosine kinase inhibitors and the p53 reactivator CP-31398. Their biological activity profile, however, was more related to sulphonamides because there was a strong cell cycle arrest in the G2/M phase. Further investigation revealed a multitargeted mechanism of the action that corresponded to the p53 protein status in the cell. Although the compounds expressed a high submicromolar activity against leukemia and colon cancers, pancreatic cancer and glioblastoma were also susceptible. Apoptosis and autophagy were confirmed as the cell death modes that corresponded with the inhibition of metabolic activity and the activation of the p53-dependent and p53-independent pathways. Namely, there was a strong activation of the p62 protein and *GADD44*. Other proteins such as cdc2 were also expressed at a higher level. Moreover, the classical caspase-dependent pathway in leukemia was observed at a lower concentration, which again confirmed a multitargeted mechanism. It can therefore be concluded that the sulfonates of quinazolines can be regarded as promising scaffolds for developing anticancer agents.

## 1. Introduction

**Figure 1:**
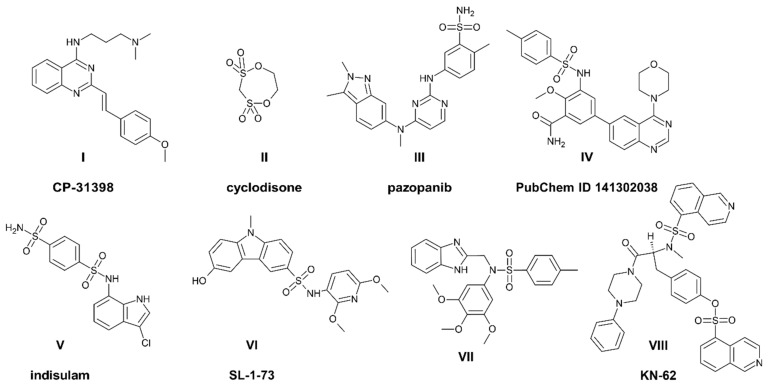
Quinazolines and sulfonyl derivatives with an anticancer activity. Structure (**I**)—reactivator of p53, known as PRIMA-1. Structures (**II**) and (**III**,**V**) are drugs used in anticancer therapy. Compounds (**IV**) and (**VI**,**VIII**) are preclinical drugs. Structure (**VII**) is potent anticancer sulfonamide described in [19].

Quinazoline has become one of the most frequently used scaffolds in medicinal chemistry and drug design. Its prevalence in natural sources and its resemblance to purines and pteridine as well as its synthetic availability strengthened our belief in the privileged nature of this motif [1,2,3]. The spectrum of activity for quinazoline derivatives is broad and covers the antiparasitic [4,5], anti-inflammatory [6], antimicrobial and antiviral [7] fields, among others. However, during the last two decades, a substantial interest has been focused on the anticancer quinazolines. Since the development of gefitinib, the first EGFR inhibitor that was approved in Japan and the USA in 2002 and 2003, respectively, worldwide interest in quinazoline-based tyrosine kinase inhibitors (TKI) has grown substantially [8,9]. However, quinazolines with an anticancer activity are not limited to TKI and also include the serine-threonine kinase inhibitors, p53 reactivators [10], folate inhibitors as well as the Wnt or Hedgehog pathway blockers [3]. Among these, the structure of 4-anilinoquinazoline is often particularly perceived as offering both a broad spectrum and high level of activity [9,11,12]. For example, 4-phenethylamine derivatives have been described as inhibitors of NF-kappa B activation [6]. In our team, several quinazoline derivatives were investigated as antimicrobial [13] and anticancer [14] agents. More recently, we investigated the p53 stabilizing agent CP-31398 (Figure 1I) as a foundation for styrylquinazoline multi-kinase inhibitors [15]. During our work on 4-anilinoquinazolines, we found that its synthetic availability through tosylates is more effective than the direct substitution of 4-chloroderivative, which is used more often in the literature [16,17,18]. Moreover, the intermediate tosylates that were generated during the synthetic procedure were resistant to hydrolysis in a water environment, which prompted us to test their activity on a panel of cancer cells. Our results became increasingly interesting as tosylate was not only active at the submicromolar level but also had a good selectivity profile and a specific mechanism of action.

Generally, sulfonic esters are commonly exploited as substrates/leaving groups in organic synthesis. The para-toluenesulfonic moiety, which is prone to nucleophilic displacement reactions, is often used in various substitution reactions. Sulfonic acids are used for counter-ions in drug development and for obtaining pharmaceutically acceptable forms with the desired solubility, stability and advantages in processing [20]. By contrast, the intentional use of these fragments in the design of bioactive substances such as drugs is rather unusual. The small molecule alkyl esters of sulfonic acids are known for their genotoxic properties, and their residual level in an end product is strictly limited. For example, the presence of ethyl methanesulfonate (EMS) resulted in the suspension of the authorization for nelfinavir mesylate by the European Medicines Agency in 2007 [21]. However, when substituted with the more resistant aryl molecule, sulfonic acid esters may afford an interesting group bioisosteric to the carboxyl or sulfonamide groups. Cyclodisone (Figure 1II) is a sulfone that should be mentioned when discussing anticancer drug design. This cyclic dithiepane derivative has been investigated as a DNA poison that forms a DNA interstrand and DNA–protein crosslinks, which is a more typical activity for the small molecular aliphatic sulfones [22,23]. Other aliphatic sulfones that should be mentioned here are treosulfan and busulfan. Both of them are based on butane derivatives and are used as typical alkylating agents in preparation for bone marrow transplantations as their anticancer administration was limited by newer, less toxic drugs.

Unlike sulfonates, sulphonamides have become increasingly popular in medicinal chemistry since they were introduced by G. Domagk in 1935 [24]. They have evolved from antibacterial chemotherapeutics to a selective anticancer agent, of which the kinase inhibitor pazopanib (Figure 1III) is a prime example. Although the sulphonamide group is in pazopanib, which is an ornamentation of the terminal phenyl ring, its importance has been revealed via its interaction with the target vascular endothelial growth factor receptor (VEGFR) [25]. The sulfonamide derivatives of 6-phenyl-quinazoline (Figure 1IV) have been described as being effective inhibitors of PI3K kinase with a similar level of activity as dactolisib [26]. Its anticancer activity has been tested on a few cancer cell lines, which confirmed its potential in a micromolar range (i.e., IC_50_ = 2.18 μM on A549 cells). Indisulam (E7070; Figure 1V) is a bisulfonamide compound that interacts with cellular dehydrogenases and destabilizes the mitochondrial redox control system, which causes cell cycle arrest in the G1 phase and a delay in the G1/S cycle [27]. Recently, a second phase of clinical trials for indisulam ended with a good overall outcome for patients with leukemia that had a strong treatment history [28]. Some structural resemblance can also be found in the carbazole [29] or benzimidazole [19] sulfonamide derivatives (Figure 1VI,VII) that have recently been published. Both classes of these compounds caused cell cycle arrest in the G2/M phase and apoptosis at low concentrations. An interesting combination of sulfonate and sulfonamide is KN-62 (Figure 1VIII), which is an inhibitor of Ca^2+^/calmodulin-dependent kinase type II (CaMKII). CaMK is a class of enzymes that are activated by elevated levels of Ca^2+^ and are therefore responsible for promoting signaling in a cell [30]. The first reports highlighted the importance of CaMKII in learning and memory functions, while some benefits were also observed in research on the cardiac physiology. Later, connections between CaMKII and cancer progression as well as its diagnosis and treatment options were also highlighted [31].

In the present work, we propose the styrylquinazoline tosylates as potential scaffolds for designing anticancer agents. As was mentioned earlier, when screening our intermediate sulfonate esters, we found some good antiproliferative activity against various cell lines. Further investigation revealed an inhibitory activity on the tyrosine kinases and a strong blocking activity on the cell cycle in the G2/M phase. A more in-depth mechanism was studied in order to reveal its metabolic reaction on sulfonates, which were found to be active. These results confirmed a strong anticancer activity especially against leukemia and brain cancer. Thus, quinazoline sulfonates can be used as new, easily accessible scaffolds for developing potent anticancer agents.

## 2. Results and Discussion

### 2.1. Synthesis of Sulfonic Styrylquinazolines

As was mentioned earlier, our approach was primarily focused on the styrylquinazoline derivatives. During the synthetic works we obtained several sulfonic esters of 4-quinazolines that were tested and evaluated for their antiproliferative potency. The general synthesis pathways of the 1,3-benzodioxol-5-sulfonate (**BS1-2**) and 4-methylbenzenesulfonate (**BS3-6**) derivatives are presented in Section 3.

**Figure 2:**
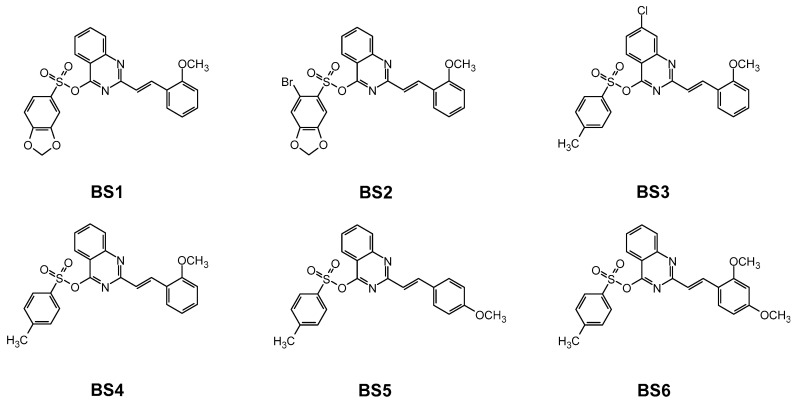
Structures of the studied compounds.

The structures of the novel compounds (Figure 2) were confirmed using ^1^H and ^13^C NMR spectroscopy and mass spectrometry, which is presented in the Supporting Information.

### 2.2. Sulfonates Demonstrate Antiproliferative Activity toward Cancer Cell Lines

The ability of the newly synthesized compounds to inhibit the proliferation of the human cancer cells was verified on a panel of seven cell lines with different genetic and protein profiles. We focused on leukemia (K562), the colon wild type (HCT 116 p53^+/+^) and one with a deletion of the *TP53* gene (HCT 116 p53^−/−^), breast (MCF-7), lung (A549), glioblastoma (U-251) and pancreatic (PANC-1) cancers. This panel represents some of the most common types of cancers, and glioblastoma (GBM) and pancreatic adenocarcinoma are extremely aggressive types of solid tumors with a poor prognosis and a five-year survival rate of less than 5%. In order to characterize the selectivity towards normal cells, we performed the experiments with healthy fibroblasts (NHDF) and calculated the selectivity index (SI) (Supporting Information Table S1). As is presented in Table 1, most of the synthetized compounds showed a good activity against the different types of cancers with only a few exceptions. The K562 cells were the most vulnerable to the tested compounds, which can at least be partly explained by their non-adhesive nature. For this cell line, we detected the lowest IC_50_ value (0.078 µM) for **BS3** with a chloride substituent in the quinazoline ring that belongs to the 4-methylbenzenesulfonate group. This activity was almost two-fold higher than the reference imatinib and 40-fold higher than CP-31398. For the rest of the derivatives, the IC_50_ values were below 0.3 µM (0.172 µM for **BS1**, 0.173 µM for **BS4** and 0.246 µM for **BS2**) or below 3 µM (2.699 µM for **BS6**). The **BS5** derivative was moderately active (IC_50_ = 10.19 µM) on the K562 cells. Generally, this 4-methylbenzenesulfonate derivative with a p-methoxy substituent in the phenyl ring of the styryl moiety had the lowest activity for the other cell lines that were tested (IC_50_ > 25 µM). Moreover, for the K562 cell line, we calculated the highest SI values: 144.51 for **BS4** and 120.71 for **BS3**. For the rest of the active compounds, the SI values were high as well: 72.91 for **BS1** and 63.70 for **BS2**. No significant differences in the activity between the HCT 116 p53^+/+^ and HCT 116 p53^−/−^ cell lines were detected, which may suggest the p53 independent mechanism of action. The colon cancer cell lines were second in their susceptibility to the tested compounds. For four derivatives (**BS1**, **BS2**, **BS3**, **BS4**), the IC_50_ values were in the range of 0.239 µM (**BS3**) to 3.724 µM (**BS4**) and had high SI values (in the range of 13.06–25.94). As was mentioned above, the p-methoxy substituent in the phenyl ring of the styryl moiety (**BS5**) had a negative influence on the activity. A double substitution of the methoxy substituent in the ortho and para positions (**BS6**) also did not improve the activity. For the PANC-1 cell lines, the second IC_50_ value (0.097 µM) for **BS3** was also detected. This derivative also had a low toxicity towards the normal cells (SI = 97.06). Generally, the ortho substitution of the methoxy group in the phenyl ring of the styryl moiety seems to be crucial for the activity. Very similar IC_50_ values (range 1.757 µM to 2.303 µM) were obtained for the U-251 cell line for the four derivatives with the o-methoxy substitution. The breast cancer cell line (MCF-7) was rather resistant to the tested compounds. The lowest IC_50_ value was 4.599 µM for **BS3**. The A549 cell line was the least vulnerable to the tested compounds. Only two representatives of the 4-methylbenzenesulfonate group, the **BS3** and **BS4** derivatives, expressed a moderate activity (IC_50_ = 7.65 µM and 11.34 µM, respectively). Generally, **BS3** was the most active compound on all of the tested cell lines. It is worth noting that most of the active compounds were more active than the reference drugs, CP-31398 and imatinib.

### 2.3. Sulfonates Modulate Tyrosine Kinases Activity

As was mentioned above, the sulfonic derivatives had an inhibitory potential against the serine-threonine or lipid (PI3K) kinases. Additionally, in our previous work, we described a novel series of 2-styrylquinazolines that had been designed as CP-31398 analogs, which showed a significant ability to inhibit a panel of non-receptor tyrosine kinases [15]. Therefore, in this work, we also decided to examine the effect of all of the tested derivatives on their activity on the ABL, BRK and BTK enzymes as well as five kinases from the Src family. Generally, when the 4-tosylates derivatives were applied at a concentration of 0.5 µM, they had moderate or weak inhibitory properties. Interestingly, all of the compounds interacted with the BTK and Lck kinases in an inhibition range from 9% to 43% (see in Table 2). It is worth noting that oncogenic BTK kinase is highly expressed in leukemia cells, which may explain the susceptibility of these cells to the tested compounds to some degree [32,33]. Moreover, several reports have shown that BTK kinase is an important element of the cascade of the signal transduction from BCR-ABL through Lyn and Syk, which leads to calcium release, cell proliferation and the activation of the NF-κB pathway [34,35]. However, it is surprising that only one compound (**BS1**) was able to inhibit the ABL enzyme, which plays a key role in CML development. On the other hand, it is worth mentioning that recent studies have also shown BTK kinase might become a major target in glioblastoma therapy [36,37]. Moreover a high expression of Lck kinase is involved in regulating the processes of glioblastoma cell migration as well as tumor survival and growth [38].

However, at a concentration of 0.5 μM, which is the level of activity for the active compounds, only a partial kinase inhibition was observed (Table 2). Therefore, it can be concluded that although our results indicate a high anticancer activity of the tosylate derivatives on the U-251 cell line, it seems that their mechanism does not rely on an interaction with the kinases. Therefore, we are convinced that the obtained derivatives have a different, more complex molecular mechanism of action, which we decided to investigate on two cell lines: leukemia (K562) and glioblastoma (U-251).

### 2.4. Sulfonates Inhibit Cell Cycle Progression

**Figure 3:**
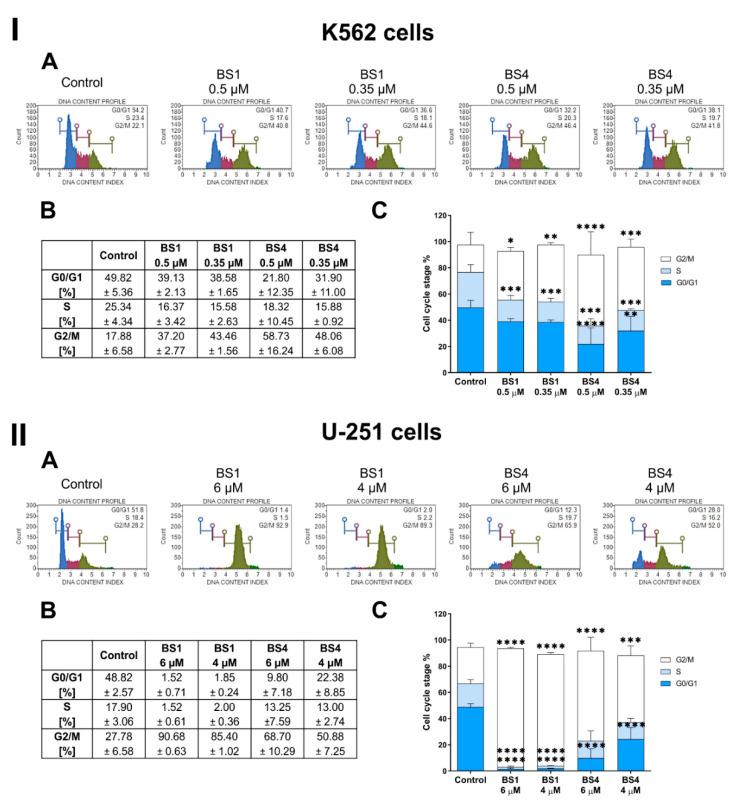
Effect of the treatment with the selected compounds (**BS1**, **BS4**) at various concentrations on regulating the cell cycle in the K562 (**I**) and U-251 (**II**) cells. The representative histograms with the distribution of the cells in the respective phases of their cycles for one of several independent experiments (**A**). The table includes the mean ± SD percentage of the cells in the respective phases of the cell cycle from all of conducted experiments (**B**). Data chart with the statistical analysis using a one-way ANOVA with Bonferroni’s post-hoc test: * *p* < 0.05, ** *p* < 0.01, *** *p* < 0.001, **** *p* < 0.0001 compared to the untreated cells (control) (**C**).

We selected two candidates for a more in-depth analysis of the molecular mechanism of action of the sulfonic styrylquinazoline derivatives. **BS1** was selected from the 1,3-benzodioxol-5-sulfonate group and **BS4** as the 4-methylbenzenesulfonate derivative. Both are characterized by one of the highest anticancer activities among the tested compounds as well as a high selectivity against normal cells. In the first step, we analyzed the cell cycle distribution in the K562 and U-251 cells using a flow cytometer after a 24 h treatment with the **BS1** and **BS4** derivatives at two different concentrations. As is presented in Figure 3, we observed cell cycle arrest in the G2/M phase in both of the tested cancer cell lines after incubation with the 4-tosylates. However, the susceptibility of the cells and the strength of the response to these agents were slightly different. Namely, for the K562 cell line, we noticed that **BS1** at 0.35 µM caused a statistically significant increase in the percentage of cells in the G2/M phase from 27.78% (untreated control cells) to 43.46%, which was accompanied by a decrease in the S and G0/G1 DNA content. Interestingly, the higher concentration of **BS1** caused a smaller increase in the percentage of cells arrested in the G2/M phase (from 27.78% to 37.20%). In turn, the second tested derivative (**BS4**) caused a significant increase in the fraction of G2/M cells in a concentration-dependent manner. The greatest effect was observed for that had been cells exposed to 0.5 µM **BS4**, while the percentage of cells in the G2/M phase increased to 58.73%. As was expected, there was a decrease in the population of cells in the G0/G1 and S phases to 21.80% (from 49.82% in the control cells) and 18.32% (from 25.34% in the control cells), respectively.

A similar but more profound behavior was observed in U-251, the second analyzed cell line. As is presented in Figure 3II(B), the percentage of the cells that were arrested in the G2/M phase increased in a concentration-dependent manner for both sulfonic derivatives. For example, at 6 µM, **BS1** caused a remarkable accumulation of cells in the G2/M phase to 90.68% (from 27.78% in the untreated cells), which was accompanied by a significant decrease in the number of cells in the G0/G1 and S phases to 1.52% (from 48.82% and 17.90% in the control cells, respectively). Interestingly, the percentage of cells in the G2/M phase was still very high (85.40%) when the U-251 cells were treated with the same derivative at a 4 µM concentration. It is noteworthy that the observed strong cell cycle arrest effect in the G2/M phase by the **BS1** derivative is greater than that of the antimitotic drug paclitaxel (Figure S19 in the Supporting Information). Our experiments showed that paclitaxel in a wide range of concentrations (0.13 µM to 2 µM) caused a strong increase in the fraction of cells in the G2/M phase to about 69.9% (from 32.3% in untreated cells). In turn, for **BS4**, there was a significant increase in the fraction of cells in the G2/M phase of about 40.92% and 23.1% (compared to the control) at a 6 µM and 4 µM concentration, respectively.

**Figure 4:**
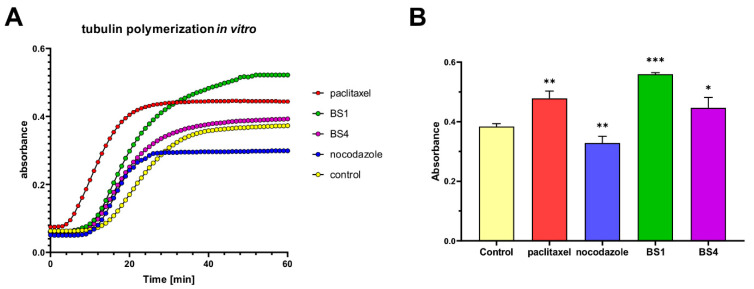
Impact of the tested sulfonic styrylquinazoline (**BS1** and **BS4**), paclitaxel and nocodazole at a concentration of 10 µM on tubulin polymerization in vitro (**A**). Data chart with maximum value absorbance of each compound with the statistical analysis using a one-way ANOVA with Bonferroni’s post-hoc test: * *p* < 0.05, ** *p* < 0.01, *** *p* < 0.001 compared to the control (**B**).

As was mentioned earlier, sulfonamide and sulfonic fragments are often used to design novel anticancer agents to improve their biological effectiveness. Among them, several derivatives have shown interesting abilities for inhibiting carbon anhydrase, the HIF-1 pathway and the antimitotic properties against the cell cycle proteins and tubulin [39,40,41,42]. Interestingly, Shaik et al. also reported that quinazoline derivatives were microtubule-disrupting agents [43]. Our results may suggest an interaction with the cell cycle proteins such as the CDKs, cyclins, Auroras, which may cause a strong cell cycle arrest in the G2/M phase. Moreover, the cell cycle arrest may be possibly, if not exclusively, associated with the interaction with microtubules. This, in turn, changes the dynamics of their assembling, which causes the observed cytotoxic effects. With this in mind, we performed in vitro tubulin polymerization assay with **BS1**, **BS4** and reference drugs: paclitaxel (enhancer of tubulin polymerization) and nocodazole (inhibitor of tubulin polymerization). As is presented in Figure 4, we observed that both tested sulfonate derivatives can influence on tubulin polymerization process. In particular, the **BS1** derivative caused a significant enhancement of tubulin polymerization (increase about 47% in compare to control). Interestingly, the observed effect was much stronger than that of the well-known enhancer of this process, paclitaxel.

Although antimitotic mechanism of action of paclitaxel is well established, it can also cause G1 arrest at low concentrations [44]. This can be partially connected with metabolic checkpoints arrest that is triggered downstream the mTOR pathway [45,46]. Glutamine deprivation was proposed as mechanism of G1 or S-phase arrest. Interestingly no similar effect can be observed in case of **BS1** or **BS4**. These observations reveal also potentially valuable direction of further investigation of use benzenesulfonates in combination with other cell cycle targeted drugs.

### 2.5. Sulfonates Induce Apoptosis and Autophagy

**Figure 5:**
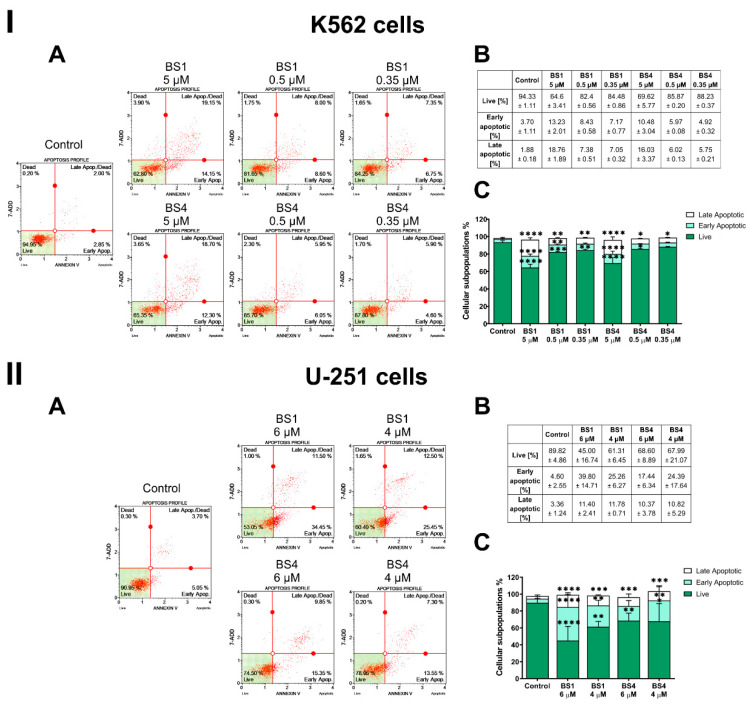
Assessment of the effect on the induction of apoptosis in the K562 (**I**) and U-251 (**II**) cells after a 48 h incubation with the tested compounds (**BS1**, **BS4**) at various concentrations. The representative histograms from one of several independent experiments include the percentage of live and apoptotic cells (**A**). The table contains the mean ± SD percentage of the live, early and late apoptotic cells from all of conducted experiments (**B**). Data chart with the statistical analysis using a one-way ANOVA with Bonferroni’s post-hoc test: * *p* < 0.05, ** *p* < 0.01, *** *p* < 0.001, **** *p* < 0.0001 compared to the untreated cells (control) (**C**).

In further experiments, we decided to explore the type of cell death that was induced by derivatives that were tested. Firstly, we performed apoptosis assays, which were based on the quantitative measurements of the green fluorescence that was emitted by the damaged cells, which had previously been labeled with FITC dye conjugated with the Annexin V protein. This protein has a high affinity for phosphatidylserine, which is translocated from the inner face of the plasma membrane to the cell surface soon after the initiation of apoptosis. In the leukemia cells, the percentage of apoptotic cells after they had been exposed to **BS1** and **BS4** increased in a concentration-dependent manner (Figure 5I). However, the population of cells in which the apoptosis process was initiated was relatively low. For the highest applied concentration of the tested tosylates, 5 µM, we observed a significant increase in the number of total (early and late) apoptotic cells from 5.56% (in the control cells) to 31.99% for **BS1** and 26.51% for **BS4**. In turn, for the lowest concentrations of both compounds (0.5 µM and 0.35 µM), the percentage of the total apoptotic cell population did not exceed 16%.

On the other hand, we registered a slightly different situation for the glioblastoma cells (Figure 5II). The highest effect was observed when cells were exposed to **BS1** at a 6 µM concentration, in which there was a significant increase the percentage of early apoptotic cells to 39.80% (from 4.60% in the untreated cells) and late apoptotic cells to 11.40% (from 3.36% in the control cells). The second analyzed derivative at 6 µM caused a significant increase in the percentage of early apoptotic cells to 17.44% and late apoptotic cells to 10.37%. A very similar effect was observed for a lower concentration.

Importantly, the effect of antimitotic drugs on the disruption of organization and stabilization of tubulin can induce a mitotic catastrophe. Additionally, a prolonged state of mitotic arrest may lead to cell death via a p53-dependent apoptosis, which is associated with the mitochondria signaling pathway [47].

**Figure 6:**
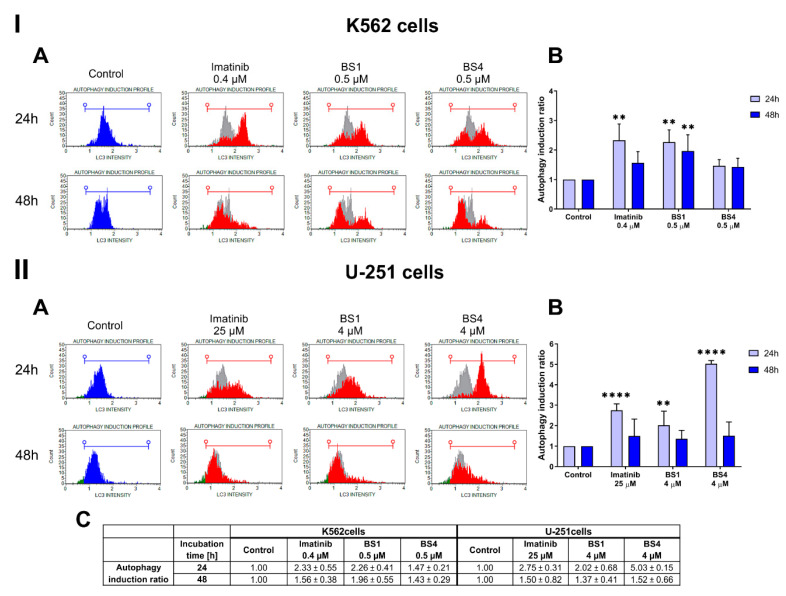
Assessment of the effect on inducing autophagy in the K562 (**I**) and U-251 (**II**) cells after a 24 and 48 h incubation with the tested compounds (**BS1**, **BS4**) at various concentrations. The representative histograms from one of several independent experiments include the autophagy induction profile (**A**). The autophagy induction ratio chart with the statistical analysis using a one-way ANOVA with Bonferroni’s post-hoc test: ** *p* < 0.01, **** *p* < 0.0001 compared to the untreated cells (control) (**B**). The table contains the mean ± SD percentage of the autophagy induction ratio from all of conducted experiments (**C**).

The interesting differences in the behavior of the compounds and the degree of behavior induction of apoptosis on the studied cell lines prompted us to consider the possibility of activating another type of cell death. Additionally, several reports have indicated that sulfonamide derivatives are able to induce cell death via autophagy [48,49]. Autophagy, like apoptosis, is regarded as being an essential mechanism in maintaining cellular homeostasis. This process is necessary for removing damaged molecular structures, organelles or protein aggregates, which enables macromolecules to be recycled in order to sustain proper cell function and metabolism [50]. In turn, in the case of long-term cellular stress or the influence of the factors that lead to continuous or excessively induced autophagy, cell death may be triggered [51,52,53]. Therefore, the pro-death and pro-survival functions of autophagy can indicate a new direction for the design of novel anticancer compounds [54]. Many reports have also indicated that inducing autophagy may be a solution to the problem of treating apoptosis-resistant cancers such as pancreatic cancers and glioblastoma [55,56]. Additionally, when autophagy is triggered, it may disrupt the chemokine-mediated migration and invasion in GBM cells [57]. To evaluate the ability of the tested 4-tosylates to induce autophagy cell death, we performed an assay, which was based on staining the cells with the anti-LC3 Alexa Fluor555 conjugated antibody. The LC3-II protein, which is generated via the conjugation of cytosolic LC3-I to phosphatidylethanolamine, which is then embedded on both sides of the autophagosomal membrane, is a characteristic marker of autophagosomes. The LC3-II expression is strictly correlated with the number of autophagic vesicles [58]. As is presented in Figure 6, patterns of autophagy induction are visible in both of the analyzed cell lines. For the leukemia cells, we recorded a statistically significant increase in the expression of the LC3 protein after 24 h and 48 h treatments with **BS1**. The calculated autophagy induction ratios were 2.26 and 1.96, respectively, compared to the control cells. A similar result was observed for the positive control, imatinib, after a 24 h incubation (ratio = 2.33). On the other hand, we observed a strong effect on autophagosome formation and autophagy induction after a 24 h treatment with both of the tested derivatives in the glioblastoma cell line. The **BS4**, for which the calculated autophagy ratio was 5.03, had the higher effect.

To summarize, these results suggest that the sulfonic derivatives have the ability to induce apoptosis and the autophagy pathways. The dual activation of programmed cell death may help to overcome multidrug resistance in several types of cancers.

### 2.6. Sulfonates Change Expression of Genes and Proteins Associated with Cell Metabolism, Cell Cycle and Cell Death

**Figure 7:**
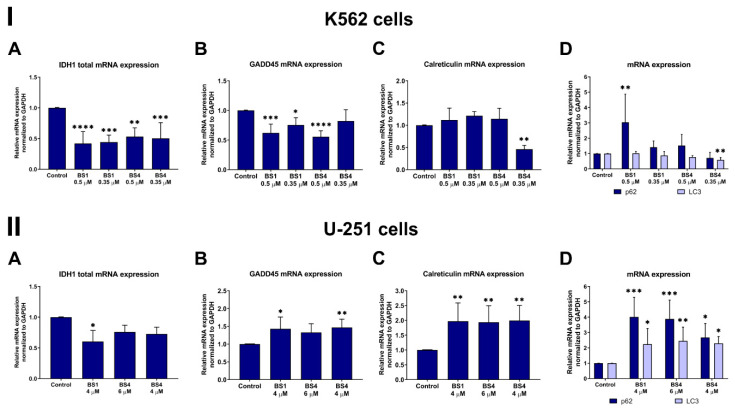
mRNA expression of selected genes, *IDH1* (**A**), *GADD45* (**B**), *calreticulin* (**C**), *p62* and *LC3* (**D**), in the K562 (**I**) and U-251 (**II**) cells after incubation with tested compounds (**BS1**, **BS4**) at various concentrations. Data are shown as the mean ± SD of several independent measurements. The statistical analysis was performed using a two- (**A**,**D**) or one-way ANOVA (**B**,**C**) with Bonferroni’s post-hoc test: * *p* < 0.05, ** *p* < 0.01, *** *p* < 0.001, **** *p* < 0.0001 compared to the untreated cells (control).

**Figure 8:**
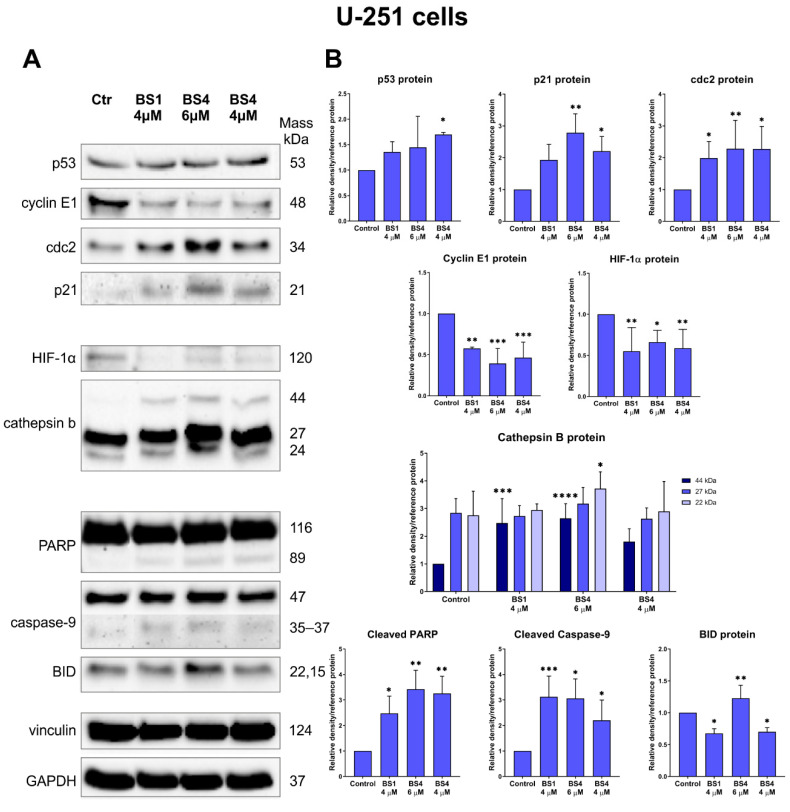
Impact of the tested derivatives (**BS1**, **BS4**) at various concentrations on the expression of selected proteins that are associated with cell cycle, apoptosis and oxidative stress in the U-251 cells (**A**). The densitometric analysis charts of obtained results were normalized to the reference protein and are presented as the mean ± SD of several independent experiments. The uncropped Western blots have been shown in Figure S22. (**B**). The statistical analysis was performed using a one-way ANOVA with Bonferroni’s post-hoc test: * *p* < 0.05, ** *p* < 0.01, *** *p* < 0.001, **** *p* < 0.0001 compared to the untreated cells (control, Ctr).

**Figure 9:**
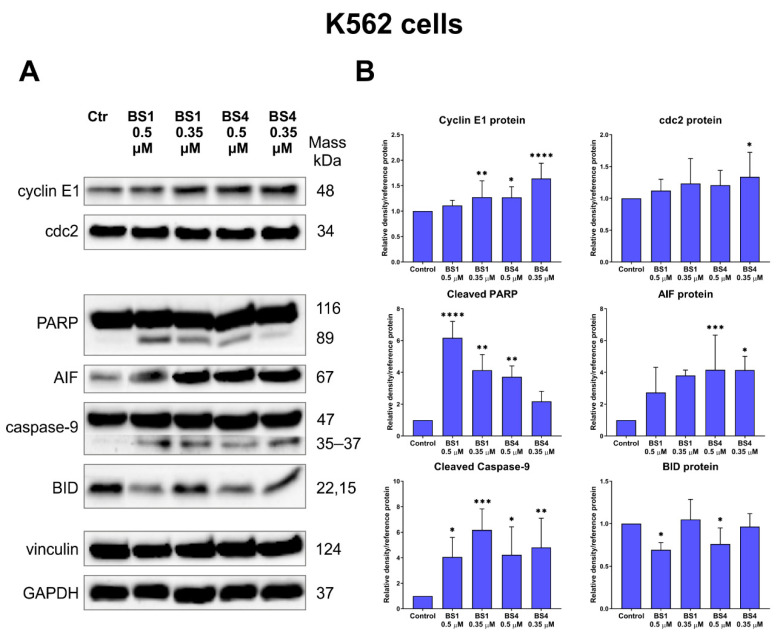
Impact of the tested derivatives (**BS1**, **BS4**) at various concentrations on the expression of selected proteins that are associated with the cell cycle and apoptosis in the K562 cells (**A**). The densitometric analysis charts of the obtained results were normalized to the reference protein and are presented as the mean ± SD of several independent experiments. The uncropped Western blots have been shown in Figure S22. (**B**). The statistical analysis was performed using a one-way ANOVA with Bonferroni’s post-hoc test: * *p* < 0.05, ** *p* < 0.01, *** *p* < 0.001, **** *p* < 0.0001 compared to the untreated cells (control, Ctr).

To further clarify the molecular mechanism of action of the sulfonic styrylquinazolines, we examined their influence on the mRNA and protein expression associated with cell metabolism, progression of cell cycle and cell death induction via apoptosis and autophagy. The results from the qRT-PCR experiments are presented in Figure 7, and the immunoblotting in Figure 8 and Figure 9. At the mRNA level, we primarily investigated the effect of the tested tosylates on altering the expression of *IDH1*, *GADD45*, *calreticulin*, *LC3* and *p62*. In turn, at the protein level, we mainly explored the cell cycle (p53, p21, cyclin E1, cdc2) and apoptosis (BID, caspase, PARP) targets.

The qRT-PCR analysis revealed that the tested compounds caused a statistically significant decrease in the expression of *IDH1* in the K562 cells (Figure 7I(A)). In detail, we observed an almost 2.4-fold decrease in the *IDH1* level for **BS1** at 0.5 µM and about a two-fold decrease for **BS4** (0.5 µM and 0.35 µM). In the glioblastoma cells, there was a decrease in the *IDH1* mRNA expression, although to a lesser extent. The change in this transcript’s level was related to the influence of the sulfonic styrylquinazolines on the overall cellular metabolism and the inhibition of cell proliferation and growth. This is supported by several reports, which revealed the potency of the sulfonamide analogs as mutant IDH1 or dual IDH1/ABL inhibitors [32,59,60]. It is worth noting that a decreased IDH1 expression may prevent the overproduction of onco-metabolites, which can usually positively regulate the HIF pathways that are involved in angiogenesis, invasion and metastasis [61]. Our results showed that both **BS1** and **BS4** caused a significant decrease in the expression of the HIF-1α protein in the glioblastoma cells (Figure 8). In turn, in the leukemia cells, the protein did not activate and was undetectable when the Western blot method was used.

The tested sulfonic styrylquinazoline derivatives contributed to cell cycle arrest in the G2/M phase. In the glioblastoma cell line, this effect was much greater than in the K562. To a large extent, this behavior is desirable due to the brisk mitotic activity, the high rate of tumor growth, the abnormal activity of the cell division regulators and, lastly, the genome instability that characterizes the GBM cells [62,63]. One of the key molecular nodes between several critical pathways including the aforementioned cell cycle as well as apoptosis, DNA repair and cellular senescence is the p53 protein. It is known as the guardian of the genome and is essential for normal cellular homeostasis and for maintaining genome integrity. It is worth mentioning that the U-251 cells contain a missense point mutation in the *TP53* gene, which results in a change of arginine into histidine at codon 273. Therefore, the mutated p53 protein loses its tumor suppression function and can enter into new interactions with other genes and transcription regulators [64]. On the other hand, literature data indicate that the K562 cell line does not express the wild-type p53 protein. The loss of one allele and an insertion mutation in exon 5 of the second allele produces a truncated form of the p53 protein with 148 amino acids [65]. This mutated form inactivates p53 and can lead to the development of drug resistance to treatment as well as to the suppression of apoptosis and progression into the blastic phase [66]. With this in mind, we determined the influence of the two tested tosylates on the p53 expression and its downstream targets. For the U-251 cells, there was a statistically significant increase in the p53 levels after the treatment with **BS4**. Interestingly, a similar styrylquinazoline, CP-31398, exhibited a potency in restoring the sequence-specific DNA-binding ability of the p53–273H mutant, which induced a pro-apoptotic response [67]. In turn, the second analyzed derivative, **BS1**, caused a slight increase in the expression of this protein. In the leukemia cells, there was no p53 activation as was expected. These results suggest that the p53-dependent and p53-independent mechanisms of apoptotic cell death pathway are activated by the sulfonic styrylquinazolines.

Other important molecules that interact with the p53 protein in cell cycle arrest in the G2/M phase are GADD45 and p21^CIP/WAF1^. As can be assumed, due to the differences in the p53 protein activation, the behavior of the U-251 and K562 cells that had been incubated with the tested derivatives was quite different. Namely, at 4 µM both **BS1** and **BS4** caused an almost 1.5-fold increase in the *GADD45α* expression in the glioblastoma cells (Figure 7). The situation was reversed for the leukemia cells, in which there was an almost two-fold decrease in the *GADD45α* mRNA expression for **BS4** at the higher concentration of 0.5 µM. A similar result was recorded for **BS1** at 0.5 µM, while much smaller differences were observed for both compounds at lower concentrations. As was mentioned above, GADD45 may be regulated by the p53 protein and plays a key role in the G2/M checkpoint in response to DNA damage. Several reports have indicated that GADD45 may inhibit the nuclear cyclin B1 protein expression, which is correlated with the activity of the cdc2/cyclin B1 complex as well as the cdc2 activity itself [68]. Surprisingly, our results showed an increase in the cdc2 protein expression after the incubation with 4-tosylates. A strong effect was observed for both compounds in the U-251 cells in which the cdc2 levels were increased by more than two-fold (Figure 8). There was a significantly enhanced cdc2 level in the K562 cells, which was responsible for entering mitosis after incubation with **BS4** at 0.35 µM (Figure 9). One explanation for this phenomenon may be related to the disorganization and damage to the microtubules that can be caused by the factors that stabilize or destabilize their polymerization. For example, Chadebech et al. indicated that an enhancer of tubulin polymerization, paclitaxel, may increase the cdc2 levels and prevent cyclin B degradation [69]. Another nocodazole may induce mitotic prometaphase arrest by up-regulating the cdc2 levels [70]. Moreover, prolonged mitotic arrest and cdc2 accumulation have also previously been reported elsewhere [71]. In addition, both GADD45 and p53 can interact with the p21^CIP/WAF1^ protein, which plays a dual role in determining cell fate. Several reports have indicated that an increase in the p21 accumulation may lead to the activation of apoptosis signaling via p53-dependent or p53-independent pathways [67,72]. Moreover, Zhong et al. revealed that the induction of cell cycle arrest in the G2/M phase by the known CP-31398 was attributable to the p21 activation [73]. In our experiments, an enhancement of the p21 level was only observed in the U-251 cells (Figure 8). The greatest effect was observed for the **BS4** compound, which caused a more than two-fold increase in its expression. In the leukemia cells, the p21 protein level was undetectable when the Western blot technique was used. On the other hand, there was a significant activation of the cyclin E1 protein in the K562 cells after the incubation with **BS1** and **BS4** at 0.35 µM (Figure 9). Contrarily, we recorded an almost two-fold decrease in the expression of cyclin E1 in the U-251 cell line after treatment with both derivatives (Figure 8). It is worth noting that modulating the cyclin E1 expression leads to cell cycle arrest or triggers apoptosis as has been reported elsewhere [51,74].

To confirm our earlier results from the autophagy experiments using flow cytometry, we further explored three other molecules *LC3*, *p62* and *calreticulin* at the mRNA level. As is shown in Figure 7, we observed a more than two-fold increase in the expression of *LC3* in the U-251 cells after treatment with both the **BS1** and **BS4** derivatives. In the second analyzed cell line, although there was no increase in the *LC3* transcript levels, the *LC3* levels decreased after the incubation with **BS4** at 0.35 µM. The situation was different for the *p62* gene, which was activated in both cancer cell lines. Namely, we recorded an almost four-fold increase in the *p62* expression when the U-251 cells were exposed to 4 µM **BS1** and 6 µM **BS4**. In those leukemia cells, there was a similar increase (about three-fold) in the *p62* level after incubation with **BS1** at 0.5 µM. These changes may be related to the extremely highly dynamic autophagic process that is initiated by the interplay of the PI3K/Beclin1 complex, Atg5–Atg12 and LC3-phosphatidylethanolamine, which results in the formation of an autophagosomal vesicle after which the p62 receptor, whose role is to selectively target and shuttle proteins/organelles into the autophagosome, is activated [75,76]. In addition, the autophagosome formation may be enhanced by the interaction of calreticulin with the LC3 [77]. Our results show more than a two-fold increase in the *calreticulin* levels in the U-251 cells after incubation with both tosylates. By contrast, there were no changes in the *calreticulin* expression in the K562 cell line, except for incubation with **BS4** at 0.35 µM. However, these results might correlate well with the greater autophagy induction in glioblastoma than in the leukemia cells.

In the final phase of autophagy, the autophagosome is fused with the lysosome, which results in the degradation of any unwanted material by the hydrolases. Inside the lysosomes, cathepsin b, which is normally involved in autophagy, occurs due to the acidic environment. However, when released from the lysosome into the cytoplasm, it can activate the apoptotic pathway through a BID cleavage and cytochrome c release from the mitochondria [78]. Surprisingly, both 4-tosylates increased the accumulation of pro-cathepsin b (44 kDa) in the U-251 cells but did not modify the expression level of cathepsin b (27 and 24 kDa) (Figure 8). There were significant fluctuations in the total BID levels after incubation with **BS1** and **BS4** for both of the tested cell lines (Figure 8 and Figure 9).

Additionally, we explored the targets that are directly associated with apoptosis induction: caspase-9, AIF and PARP. Our results indicate that the cleavage of caspase-9, which initiates apoptosis, was induced after incubation with the tested derivatives in the U-251 and K562 cell lines. In detail, there was an approximately three-fold increase in the expression of the cleavage product of caspase-9 after the U-251 cells were treated with **BS1** (4 µM) and **BS4** (6 µM) (Figure 8). In the K562 cell line, the observed effect was greater; **BS1** at 0.35 µM caused a more than six-fold increase in the level of cleavage caspase-9 proteins (Figure 9). Interestingly, in the leukemia cells, there was a significant increase in the AIF protein expression, which is a caspase-independent apoptosis death effector. In the case of glioblastoma, the AIF protein was not activated. The last examined protein was PAPR, which is responsible for DNA repair as well as in transcriptional regulation or chromatin remodeling. This molecule is cleaved and inactivated by the caspases, which leads to cell disintegration followed by the activation of apoptosis. As was expected, the cleavage product of PARP was present in the analyzed U-251 and K562 cell lines after they had been incubated with **BS1** and **BS4**. To summarize, the sulfonic styrylquinazolines are capable of activating the p53-dependent or p53-independent apoptosis pathway. Additionally, they can induce apoptosis via effectors that act in a caspase-dependent or independent manner.

## 3. Materials and Methods

The ^1^H and ^13^C NMR spectra were recorded on a Brucker 500 (126 MHz) spectrometer. The chemical shifts are reported in ppm (δ) relative to TMS with the respective solvent resonance as the internal standard, (CD_3_)_2_SO δ 2.50 and 40.03 ppm, respectively. Coupling constants < 0.5 Hz were not taken into account when determining multiplicity. The samples were prepared in concentrations in the range 5–10 mM. The representative preparation protocols of the precursors were published elsewhere.

### 3.1. Synthesis

**Figure 10:**
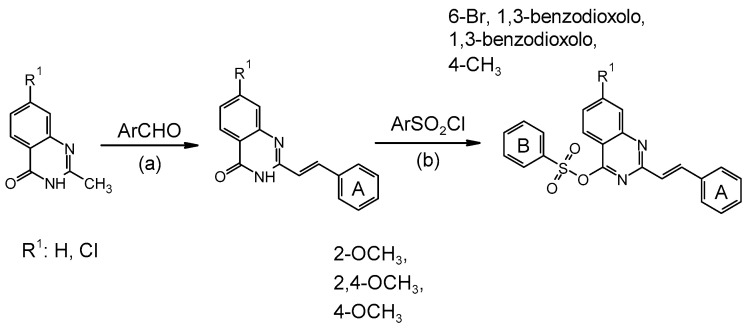
Synthesis pathways of the sulfonic derivatives of styrylquinazolines. (A) 2-OCH_3_; 2,4-OCH_3_; 4-OCH_3_; (B) 6-Br, 1,3-benzodioxolo; 1,3-benzodioxolo; 4-CH_3_. Conditions: (a) AcOH, MW 80 W, 140 °C; (b) CH_2_Cl_2_, DIPEA, DMAP, RT.

The 2-[(*E*)-2-phenylethenyl]quinazolin-4(3*H*)-one precursors were synthesized by condensing aromatic aldehydes (2 mmol) with a 2-methyl-4(3*H*)-quinazolinone core (1 mmol). The reactions were conducted in acetic acid (3 mL, 99.5%) under microwave irradiation (sealed vial, 80 W, 140 °C, 90 min) after which the vial was cooled, and the precipitate was separated via filtration and washed with 2-propanol. The target compounds were synthesized according to a modified procedure that had been reported in our previous work [15] or that had been published elsewhere [79]. Generally, *N*,*N*-diisopropylethylamine (26 mg, 0.4 mmol), benzenesulfonic chloride (0.4 mmol) and 4-dimethylaminopyridine (2 mg, 0.02 mmol) were added to a suspension of 2-[(*E*)-2-phenylethenyl]quinazolin-4(3*H*)-one precursor (0.2 mmol) in 2 mL of CH_2_Cl_2_. The reaction mixture was stirred at room temperature until the mixture had become homogenous or until the substrate had disappeared (TLC). The reactions were quenched by adding the solution to 20 mL of 2-propanol. The precipitate was collected, washed with two portions (2 mL) of alcohol and dried under a stream of argon. The general synthesis pathways of the sulfonic derivatives are presented in Figure 10.

**(BS1)**  2-((*E*)-2-(2-methoxyphenyl)ethenyl)quinazolin-4-yl 1,3-benzodioxole-5-sulfonate ^1^H NMR (500 MHz, (CD_3_)_2_SO) δ 8.26 (d, *J* = 16.2 Hz, 1H), 8.15 (dd, *J* = 8.0, 1.5 Hz, 1H), 7.89 (ddd, *J* = 8.5, 7.2, 1.6 Hz, 1H), 7.73 (dd, *J* = 8.3, 1.1 Hz, 1H), 7.63 (dd, *J* = 7.8, 1.7 Hz, 1H), 7.56 (ddd, *J* = 8.1, 7.1, 1.1 Hz, 1H), 7.47 (ddd, *J* = 8.8, 7.4, 1.7 Hz, 1H), 7.17 (d, *J* = 8.4 Hz, 1H), 7.15 (d, *J* = 16.3 Hz, 1H), 7.13 (dd, *J* = 8.0, 1.7 Hz, 1H), 7.08 (td, *J* = 7.5, 1.0 Hz, 1H), 7.04 (d, *J* = 1.6 Hz, 1H), 6.83 (d, *J* = 8.0 Hz, 1H), 6.01 (s, 2H), 3.94 (s, 3H); ^13^C NMR (126 MHz, (CD_3_)_2_SO) δ 161.16, 158.94, 154.16, 147.71, 146.93, 143.74, 143.06, 139.23, 136.01, 133.18, 129.90, 127.84, 126.88, 123.54, 123.01, 121.53, 120.59, 119.88, 117.78, 112.61, 107.61, 106.72, 101.55, 56.28; HRMS (ESI) calcd for C_24_H_18_N_2_O_6_S [M − H]^−^ 461.0813; found 461.0821; m.p. 223–225 °C.
**(BS2)**  2-((*E*)-2-(2-methoxyphenyl)ethenyl)quinazolin-4-yl 6-bromo-1,3-benzodioxole-5-sulfonate ^1^H NMR (500 MHz, (CD_3_)_2_SO) δ 8.18 (ddd, *J* = 8.3, 1.5, 0.7 Hz, 1H), 8.08 (ddd, *J* = 8.4, 6.9, 1.4 Hz, 1H), 8.01 (appdt, *J* = 8.4, 1.0 Hz, 1H), 7.92 (d, *J* = 16.1 Hz, 1H), 7.86 (s, 1H), 7.78 (ddd, *J* = 8.1, 6.9, 1.2 Hz, 1H), 7.70 (dd, *J* = 7.7, 1.7 Hz, 1H), 7.62 (s, 1H), 7.41 (ddd, *J* = 8.9, 7.3, 1.7 Hz, 1H), 7.26 (d, *J* = 16.0 Hz, 1H), 7.13 (dd, *J* = 8.4, 1.1 Hz, 1H), 7.03 (apptd, *J* = 7.5, 1.1 Hz, 1H), 6.12 (s, 2H), 3.95 (s, 3H); ^13^C NMR (126 MHz, DMSO) δ 162.00, 158.43, 152.80, 148.39, 146.40, 141.67, 135.23, 132.10, 128.99, 126.86, 126.46, 123.66, 121.39, 121.27, 113.80, 112.41, 111.15, 109.47, 102.45, 56.16; HRMS (ESI) calcd for C_24_H_17_BrN_2_O_6_S [M + H]^−^ 541.0063; found 541.0079; m.p. 149–151 °C.
**(BS3)**  7-chloro-2-((*E*)-2-(2-methoxyphenyl)ethenyl)quinazolin-4-yl 4-methylbenzenesulfonate ^1^H NMR (500 MHz, (CD_3_)_2_SO) δ 8.19 (d, *J* = 16.2 Hz, 1H), 8.09 (d, *J* = 8.5 Hz, 1H), 7.74 (d, *J* = 2.0 Hz, 1H), 7.61 (dd, *J* = 7.7, 1.9 Hz, 1H), 7.50 (dd, *J* = 8.5, 2.1 Hz, 1H), 7.49–7.46 (m, 2H), 7.43 (ddd, *J* = 8.9, 7.5, 1.8 Hz, 1H), 7.14 (d, *J* = 8.2 Hz, 1H), 7.13 –7.10 (m, 2H), 7.08 (d, *J* = 16.2 Hz, 1H), 7.04 (appt, *J* = 7.3 Hz, 1H), 3.92 (s, 2H), 2.29 (s, 3H); ^13^C NMR (126 MHz, (CD_3_)_2_SO) δ 161.64, 158.38, 153.86, 150.28, 146.25, 139.60, 138.04, 135.35, 132.05, 128.88, 128.51 (2H), 128.42, 126.76, 126.33, 125.97 (2H), 128.51, 123.65, 121.37, 121.18, 120.25, 112.38, 56.14, 21.25; HRMS (ESI) calcd for C_24_H_19_ClN_2_O_4_S [M − H]^−^ 465.0681; found 465.0685; m.p. 196–197 °C.
**(BS4)**  2-((*E*)-2-(2-methoxyphenyl)ethenyl)quinazolin-4-yl 4-methylbenzenesulfonate ^1^H NMR (500 MHz, (CD_3_)_2_SO) δ ^1^H NMR (500 MHz, DMSO) δ 8.26 (d, *J* = 16.3 Hz, 1H), 8.14 (dd, *J* = 8.0, 1.5 Hz, 1H), 7.88 (ddd, *J* = 8.5, 7.1, 1.6 Hz, 1H), 7.73 (dd, *J* = 8.3, 1.1 Hz, 1H), 7.63 (dd, *J* = 7.7, 1.7 Hz, 1H), 7.56 (ddd, *J* = 8.1, 7.1, 1.1 Hz, 1H), 7.49–7.47 (m, 2H), 7.47 (ddd, *J* = 6.9, 1.8 Hz, 1H), 7.17 (dd, *J* = 8.4, 1.1 Hz, 1H), 7.15 (d, *J* = 16.4 Hz, 1H), 7.13–7.11 (m, 1H), 7.08 (td, *J* = 7.5, 1.0 Hz, 1H), 3.94 (s, 3H), 2.29 (s, 3H); ^13^C NMR (126 MHz, (CD_3_)_2_SO) δ 161.40, 159.96, 158.05, 153.16, 146.79, 136.23, 134.66, 131.60, 130.57, 129.26, 128.99, 128.40, 128.10, 127.13, 124.08, 123.55, 121.34, 114.50, 112.24, 56.24, 21.70; HRMS (ESI) calcd for C_24_H_21_N_2_O_4_S [M − H]^−^ 431.1070; found 431.1068; m.p. 168–169 °C.
**(BS5)**  2-((*E*)-2-(4-methoxyphenyl)ethenyl)quinazolin-4-yl 4-methylbenzenesulfonate ^1^H NMR (500 MHz, (CD_3_)_2_SO) δ 8.16 (dd, *J* = 8.0, 1.5 Hz, 1H), 8.12 (d, *J* = 16.3 Hz, 1H), 7.92 (ddd, *J* = 8.5, 7.2, 1.5 Hz, 1H), 7.73 (d, *J* = 7.5 Hz, 1H), 7.69–7.64 (m, 2H), 7.60 (ddd, *J* = 8.1, 7.2, 1.1 Hz, 1H), 7.52–7.48 (m, 2H), 7.13 (dd, *J* = 8.5, 0.8 Hz, 2H), 7.10–7.06 (m, 2H), 6.91 (d, *J* = 16.3 Hz, 1H), 3.84 (s, 3H), 2.29 (s, 3H); ^13^C NMR (126 MHz, (CD_3_)_2_SO) δ 161.80, 160.20, 153.64, 145.17, 143.61, 142.35, 137.76, 135.42, 130.17, 127.95, 127.23, 126.56, 126.31, 125.36, 122.21, 119.72, 114.76, 113.19, 55.36, 20.56. HRMS (ESI) calcd for C_24_H_20_N_2_O_4_S [M + H]^−^ 433.1217; found 433.1214; m.p. 176–178 °C
**(BS6)**  2-((*E*)-2-(2,4-dimethoxyphenyl)ethenyl)quinazolin-4-yl 4-methylbenzenesulfonate ^1^H NMR (500 MHz, (CD_3_)_2_SO) δ 8.18–8.14 (m, 2H), 8.09 (ddd, *J* = 8.2, 1.4, 0.7 Hz, 1H), 8.03 (ddd, *J* = 8.2, 7.0, 1.4 Hz, 1H), 8.01 (d, *J* = 16.2 Hz, 1H), 7.95 (appdt, *J* = 8.5, 1.0 Hz, 1H), 7.72 (ddd, *J* = 8.2, 7.0, 1.2 Hz, 1H), 7.69 (d, *J* = 8.6 Hz, 1H), 7.53 (m, 2H), 7.15 (d, *J* = 16.0 Hz, 1H), 6.70 (d, *J* = 2.4 Hz, 1H), 6.65 (dd, *J* = 8.5, 2.4 Hz, 1H), 3.99 (s, 3H), 3.86 (s, 3H), 2.42 (s, 3H); ^13^C NMR (126 MHz, DMSO) δ 162.57, 161.29, 160.38, 159.53, 153.25, 146.74, 136.14, 134.75, 133.52, 130.55, 129.80, 129.23, 128.63, 127.95, 124.48, 123.52, 117.06, 114.32, 106.69, 98.96, 56.33, 55.96, 21.71; HRMS (ESI) calcd for C_25_H_23_N_2_O_5_S [M + H]^−^ 463.1322; found 463.1325; m.p. 178–180 °C.

### 3.2. Cell Culture

The human colon carcinoma cell line HCT 116 wild type (p53^+/+^), the human breast carcinoma cell line MCF-7 and the human alveolar basal epithelial cell line A549 were obtained from ATCC. The human colon cancer cell line HCT 116 with a p53 deletion (p53^−/−^) was kindly supplied by prof. M. Rusin from the Maria Sklodowska-Curie Memorial Cancer Centre and Institute of Oncology in Gliwice, Poland. The glioblastoma cell line U-251 was kindly provided by prof. G. Kramer-Marek from the Institute of Cancer Research in London, United Kingdom. The human suspension chronic myelogenous leukemia cell line K562 and pancreas ductal adenocarcinoma cell line PANC-1 were purchased from Sigma-Aldrich (St. Louis, MO, USA), while the normal human dermal fibroblasts cell line NHDF were purchased from PromoCell. All of the adherent cancer cell lines were cultured in Dulbecco’s modified Eagle’s medium (DMEM) that had been supplemented with 12% heat-inactivated fetal bovine serum–FBS (all from Sigma-Aldrich) in 75 cm^2^ flasks (Nunc). The suspension cell line K562 was cultured in an RPMI-1640 medium (Sigma-Aldrich), which contained 10% heat-inactivated FBS. The DMEM for the NHDF was supplemented with 15% non-inactivated FBS. Each medium contained a combination of two antibiotics: penicillin and streptomycin (1% *v*/*v*; Gibco). All of these cell lines were grown under standard conditions at 37 °C with a 5% CO_2_ humidified atmosphere. Moreover, all of the cell lines were routinely tested for mycoplasma using the PCR technique with specific *Mycoplasma* primers to confirm that there was no contamination.

### 3.3. Cytotoxicity Studies

The cells were seeded in 96-well plates (Nunc) at a density of 5000 cells per well (K562, HCT 116, MCF-7, U-251, A549, PANC-1) or 4000 cell per well (NHDF) and incubated under standard conditions at 37 °C for 24 h. The assay was performed following a 72 h incubation with the various concentrations of the tested compounds. Then, 100 µL DMEM without phenol red with 20 µL of the CellTiter 96^®^AQueous One Solution-MTS (Promega, Promega, WI, USA) was added to each well and incubated for 1 h or 3 h (PANC-1) at 37 °C. The optical densities of the samples were measured at 490 nm using a multi-plate reader (Synergy 4, BioTek, Winooski, VT, USA). The obtained results were compared to the control and were estimated as the inhibitory concentration (IC_50_) values (using GraphPad Prism 8). Each individual compound was tested in triplicate in a single experiment with each experiment being performed three or four times.

### 3.4. Tyrosine Kinase Assay

Assays using the Kinase Selectivity TK-2 profiling systems and ADP-Glo Kinase Assay (both from Promega) were performed to determine the inhibition of the non-receptor tyrosine kinases. The protocol was previously designed and described by our group in reports [15,80]. The experiments were performed at least four times. The data are expressed as the percentage of the inhibition activity of tyrosine kinase after treatment with the tested derivatives.

### 3.5. Cell Cycle Assay

The K562, U-251 and NHDF cells were seeded in 3 cm Petri dishes (Nunc) at a density of 250,000 cells per well and incubated under standard conditions at 37 °C for 24 h. Then, the medium was removed, and freshly prepared solutions of the tested compounds, **BS1**, **BS4** (two to three times the IC_50_ value) and paclitaxel (0.13 µM; 0.26 µM; 0.5 µM, 1 µM and 2 µM) were added. After a 24 h treatment, the assays were performed using a Muse Cell-Cycle Kit (Millipore, Burlington, MA, USA) according to the supplier’s instructions. Briefly, the cells were collected, washed with cold PBS and centrifuged at 300 g for 5 min. Afterwards, the cells were fixed in ice cold 70% ethanol and stored at −20 °C overnight. The next day, the cells were washed with cold PBS, centrifuged and resuspended in 200 μL of the Muse™ Cell Cycle Reagent. The samples were incubated for 30 min at room temperature in the dark. After staining, the cellular subpopulation values in the individual cell cycle phases were estimated using a cell cycle analysis using a Muse Cell Analyzer (Millipore). The experiments were performed at least four times.

### 3.6. Tubulin Polymerization Assay

The effect of small-molecule compounds on tubulin polymerization was monitored using an In Vitro Tubulin Polymerization Kit (≥99% Pure Bovine Tubulin) (Millipore) following the manufacturer’s protocol. Briefly, the tested compounds, **BS1** and **BS4,** as well as reference drugs, paclitaxel (enhancer of polymerization) and nocodazole (inhibitor of polymerization) at a concentration of 10 µM were mixed with purified bovine tubulin (60 μM) and polymerization buffer with GTP (1 mM) on 96-well plate on ice. Then, the plate was transferred into the multi-plate reader (Synergy 4) chamber (heated to 37 °C) and measured the turbidity variation every 30 s at 350 nm during 90 min. The experiments were performed three times.

### 3.7. Annexin V Binding Assay

The K562, U-251 and NHDF cells were seeded in 3 cm Petri dishes (Nunc) at a density of 250,000 cells per well and incubated at 37 °C for 24 h. Then, the medium was removed, and freshly prepared solutions of the tested compounds, **BS1** and **BS4** (two to three times the IC_50_ value), were added. After a 48 h incubation, the assays were performed using a FITC Annexin V Apoptosis Detection KIT with 7-AAD (Bio-Legend) according to the manufacturer’s instructions. Briefly, the cells were collected, washed twice with cold PBS and centrifuged at 300 g for 5 min. Next, the cells were resuspended in a 100 µL Annexin V Binding Buffer and incubated for 15 min at room temperature in the dark with 5 µL of FITC Annexin V and 5 µL 7-AAD Viability Staining Solution. After immunostaining, the number of events for live, early and late apoptotic cells were determined using a Muse Cell Analyzer. The experiments were performed at least four times.

### 3.8. Autophagy Assay

The K562 and U-251 cells were seeded in 96-well plates (Nunc) and incubated at 37 °C for 24 h. The density of the cells was 20,000 cells per well (for the 24 h assay) and 10,000 per well (for the 48 h assay). Next, the medium was changed for freshly prepared solutions of the tested compounds (**BS1**, **BS4**) at 0.5 µM (for K562) and 4 µM (for U-251) concentrations, and the cells were incubated for 24 h or 48 h, respectively. Additionally, the cells were treated with imatinib (0.4 µM for K562 and 25 µM for U-251) as the positive control. After treatment, the assays were performed using a Muse™ Autophagy LC3-antibody based kit (Millipore) according to the manufacturer’s instructions. Briefly, the cells were collected, washed with cold HPBS and centrifuged at 300 g for 5 min. Then, the cells were resuspended in a mixture of 95 µL 1X Autophagy Reagent B and 5 μL Anti-LC3 Alexa Fluor^®^ 555 antibody. The samples were incubated on ice for 30 min in the dark. After incubation, the cells were centrifuged and resuspended in 200 μL of a 1X Assay Buffer. Then, the samples were directly processed to analyze the autophagy induction using a Muse Cell Analyzer. The autophagy induction ratio was calculated on the basis of the ratio between the target sample fluorescence versus the control sample. The experiments were performed at least three times.

### 3.9. Analysis of the mRNA Expression

The K562 and U-251 cells were seeded in 3 cm Petri dishes (Nunc) at a density of 500,000 cells per well and incubated overnight, after which the cells were incubated with freshly prepared solutions of the tested compounds (**BS1**, **BS4**, two to three times the IC_50_ value) for 24 h. The total RNA was isolated from the K562 and U-251 cells using the TRIzol Reagent procedure (Ambion, Austin, TX, USA). Reverse transcription was performed with 5 μg of total RNA using a GoScript™ Reverse Transcriptase kit (Promega) and Oligo(dT)_23_ Primers (Sigma). Real-time PCR was performed using a CTX96 Touch™ Real-Time PCR Detection System (Biorad, Hercules, CA, USA) in a 20 μL reaction volume. The reaction consisted of SsoAdvanced™ Universal SYBR^®^ Green Supermix (Biorad), a specific primer pair mix (0.5 μM each) and 1 μL of cDNA. The reaction was performed under the following conditions: initial denaturation at 95 °C for 30 s; followed by 40 denaturation cycles at 95 °C, 15 s; annealing (primer-specific temperature for 30 s) and extension at 72 °C for 60 s. The obtained results were analyzed based on a comparison of the expression of the target genes to the reference gene, GAPDH, using the 2^−ΔΔCT^ method. The experiments were performed at least four times. All of the primer pair sequences were purchased from Sigma-Aldrich and are listed in Table S2.

### 3.10. Immunoblotting

The K562 and U-251 cells were seeded in 3 cm Petri dishes (Nunc) at a density of 500,000 cells per well and incubated overnight. Then, the cells were incubated with freshly prepared solutions of tested the compounds (**BS1**, **BS4**, two to three times the IC_50_ value) for 24 h, after which the cells were detached by trypsinization and centrifuged at 2000 rpm. Next, the cell pellets were resuspended in an RIPA buffer containing a Halt Protease Inhibitor Cocktail and a Halt Phosphatase Inhibitor Cocktail along with 0.5 M EDTA (all from Thermo Scientific, Waltham, MA, USA) and lysed on ice for 20 min. Then, the obtained lysates were sonicated and centrifuged at 10,000 rpm for 10 min at 4 °C. The supernatants were collected for further analysis. The protein concentration was measured using a Micro BCA™ Protein Assay Kit (Thermo Scientific) according to the manufacturer’s instructions. Equal amounts of the proteins (16 μg) were electrophoresed on SDS-Page gels and transferred onto nitrocellulose membranes. The membranes were blocked in 5% non-fat milk prepared in TPBS (PBS containing 0.1% Tween-20) for 1 h. After blocking, the membranes were incubated with the specific primary antibodies (all from Cell Signaling) at a 1:1000 dilution—cyclin E, cdc2, PARP, AIF, BID, p53, p21^Waf1/Cip1^, HIF-1α, cathepsin B and caspase-9—and at a 1:2000 dilution for the reference proteins—vinculin, β-actin and GAPDH—overnight at 4 °C. The next day, the membranes were washed in TPBS and incubated with horseradish peroxidase (HRP)-conjugated secondary antibodies for 1 h at room temperature. Lastly, the membranes were washed in TPBS and incubated with a SuperSignal™ West Pico Chemiluminescent Substrate (Thermo Scientific). The chemiluminescence signals were captured using a ChemiDoc™ XRS + System (Biorad). The experiments were performed at least four to five times. The densitometric analysis was conducted using ImageJ software (Wayne Rasband, National Institutes of Health, Bethesda, MD, USA).

### 3.11. Statistical Analysis

The results are presented as the mean ± standard deviation (SD) from all of the independent experiments that were conducted. The statistical analysis was performed using a one- or two-way ANOVA with a Bonferroni post-hoc test. A *p*-value of 0.05 or less was considered to be statistically significant. GraphPad Prism 8.0 software (GraphPad Software, San Diego, CA, USA) was used for analysis.

## 4. Conclusions

The sulfonate derivatives of styrylquinazolines are interesting potential scaffolds for anticancer drug design. We performed an in-depth investigation of their potential mechanism of action for a library of six different structures. The compounds had a good level of antiproliferative activity and selectivity in a panel of cancer cell lines. Among them, the tosylate of 7-chloro-2-styrylquinazoline appeared to be the most active at a sub-micromolar level and was considerably higher than both of the positive control drugs. Regardless of their structural similarity to the p53 reactivator and kinase inhibitors, the activity of the tested sulfonates seems to be unrelated to these targets. However, some of the dependence on the p53 status that was observed can be explained as a signal of a multitargeted action. There was a strong arrest in the G2/M cell cycle followed by apoptosis as the model of cell death. In glioblastoma, however, where the *TP53* mutation leads to gaining of abnormal functionality, the tested compounds led to autophagy to a greater extent. These pathways were confirmed by analyzing the resulting proteins such as GADD or the cyclin-dependent kinases. In the leukemia cell line in which the functionality of the p53 protein was lost, the sulfonates expressed a higher antiproliferative potency and a more complex multitargeted mechanism as was evidenced by the different responses to the low and high concentrations. Therefore, further research is needed to reveal the factors that are responsible for distinguishing between each of the pathways of activity. Another potentially valuable direction that deserves further investigation is combinatorial therapy of these sulfonates with other anticancer agents such as doxorubicine. This can be hypothesized that strong G2/M inhibitors may be effectively used for overcoming drug resistance or adverse effects as is known for paclitaxel.

## Figures and Tables

**Table 1:** The antiproliferative activity of the studied compounds on a panel of human cancer cell lines and normal human fibroblast cells.

No.	Activity IC_50_ Value [μM]							
	K562	HCT 116 p53^+/+^	HCT 116 p53^−/−^	MCF-7	A549	U-251	PANC-1	NHDF
BS1	0.172 ± 0.034	0.880 ± 0.086	0.563 ± 0.121	8.325 ± 1.940	>25	1.897 ± 0.649	3.981 ± 0.597	12.540 ± 0.855
BS2	0.246 ± 0.055	1.200 ± 0.132	1.312 ± 0.290	16.760 ± 2.060	>25	2.303 ± 0.234	2.905 ± 0.622	15.670 ± 1.410
BS3	0.078 ± 0.027	0.363 ± 0.028	0.239 ± 0.030	4.599 ± 1.022	7.652 ± 0.987	1.757 ± 0.388	0.097 ± 0.030	9.415 ± 1.652
BS4	0.173 ± 0.031	1.567 ± 0.357	3.724 ± 0.487	9.128 ± 2.053	11.340 ± 1.760	1.907 ± 0.214	0.235 ± 0.042	>25
BS5	10.190 ± 0.819	>25	>25	>25	>25	>25	>25	>25
BS6	2.699 ± 0.519	>25	21.670 ± 1.185	9.791 ± 1.232	>25	>25	>25	>25
CP-31398	3.087 ± 0.360	18.63 ± 0.92	26.28 ± 1.41	26.96 ± 2.10	>25	18.77 ± 1.65	>25	12.26 ± 0.54
Imatinib	0.133 ± 0.030	44.55 ± 2.41	51.21 ± 4.09	>25	>25	>25	>25	>25

**Table 2:** The anti-enzymatic activity of the tested styrylquinazolines against a panel of eight non-receptor tyrosine kinases.

No.	Inhibitory Effect of the Tyrosine Kinase Activity [%] at 0.5 μM							
	ABL1	BRK	BTK	CSK	Fyn A	Lck	Lyn B	Src
BS1	28.39	15.30	18.80	0	23.68	25.78	0	0.19
BS2	0	9.33	40.18	34.25	0	9.93	37.96	26.32
BS3	0	26.46	30.66	0	34.07	42.65	0	2.73
BS4	0	35.45	26.89	28.30	3.51	19.50	0	0
BS5	0	20.23	8.59	20.10	0	11.20	0	0
BS6	0	0	17.95	21.14	62.60	37.26	0	0
CP-31398	10.02	34.70	36.83	0.98	30.65	27.87	47.10	15.73
Imatinib	77.17	0	0	0	0	0	0	0

## Supplementary Materials

The following are available online at https://www.mdpi.com/article/10.3390/cancers13081790/s1, Figure S1: ^1^H NMR plot of **BS1**, Figure S2: ^13^C NMR plot of **BS1**, Figure S3: ^1^H NMR plot of **BS2**, Figure S4: ^13^C NMR plot of **BS2**, Figure S5: ^1^H NMR plot of **BS3**, Figure S6: ^13^C NMR plot of **BS3**, Figure S7: ^1^H NMR plot of **BS4**, Figure S8: ^13^C NMR plot of **BS4**, Figure S9: ^1^H NMR plot of **BS5**, Figure S10: ^13^C NMR plot of **BS5**, Figure S11: ^1^H NMR plot of **BS6**, Figure S12: ^13^C NMR plot of **BS6**, Figure S13: HR-ESI spectrum of **BS1**, Figure S14: HR-ESI spectrum of **BS2**, Figure S15: HR-ESI spectrum of **BS3**, Figure S16: HR-ESI spectrum of **BS4**, Figure S17: HR-ESI spectrum of **BS5**, Figure **S18**: HR-ESI spectrum of **BS6**, Figure S19: Effect of the treatment with the paclitaxel at various concentrations on regulating the cell cycle in U-251 cells, Figure S20: Effect of the treatment with the selected compounds (**BS1**, **BS4**) at various concentrations on regulating the cell cycle in NHDF cells, Figure S21: Assessment of the effect on the induction of apoptosis in the NHDF cells after a 48-hour incubation with the tested compounds (**BS1**, **BS4**) at various concentrations, Figure S22: The uncropped Western blots, Table S1: Selectivity index of tested derivatives, Table S2: Sequences of primer pairs used in determining the mRNA expression of tested genes.
