# Supplementary material for: Novel Benzenesulfonate Scaffolds with a High Anticancer Activity and G2/M Cell Cycle Arrest
Source: Cancers (Basel). 2021 Apr 9;13(8):1790. doi: 10.3390/cancers13081790 (PMC8068801; doi:10.3390/cancers13081790)
Supplement: Supplementary file 1 [file cancers-13-01790-s001.pdf]

cancers

MDPI

# Supplementary Materials: Novel Benzenesulfonate Scaffolds with a High Anticancer Activity and G2/M Cell Cycle Arrest

Katarzyna Malarz, Jacek Mularski, Michał Kuczak, Anna Mrozek-Wilczkiewicz and Robert Musiol

## 1. Chemistry Data

### $^{1}$H and $^{13}$C NMR spectra of target compounds:

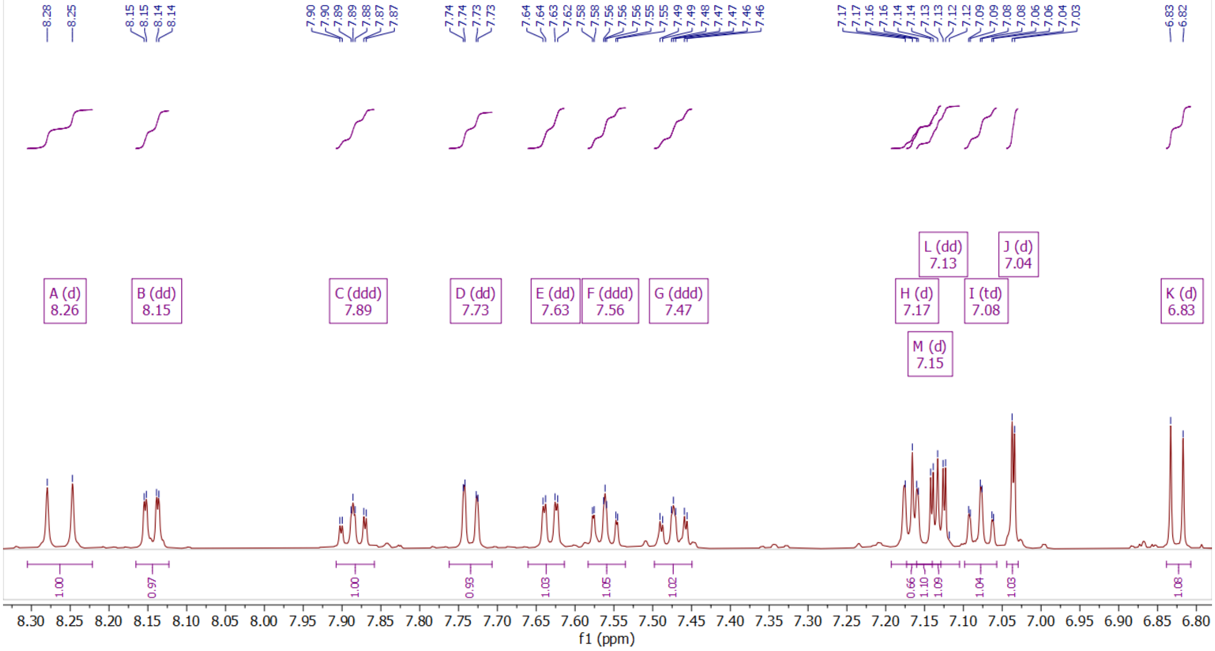


**Figure S1.** $^{1}$H NMR plot of **BS1**.

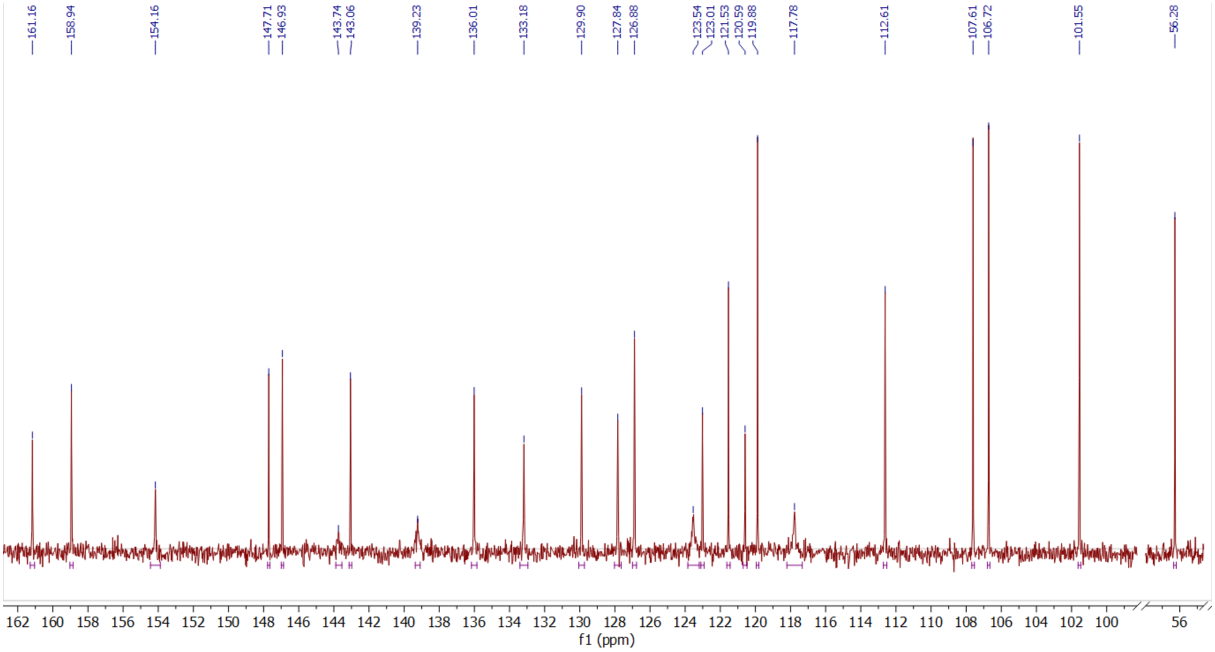


**Figure S2.** $^{13}$C NMR plot of **BS1**.

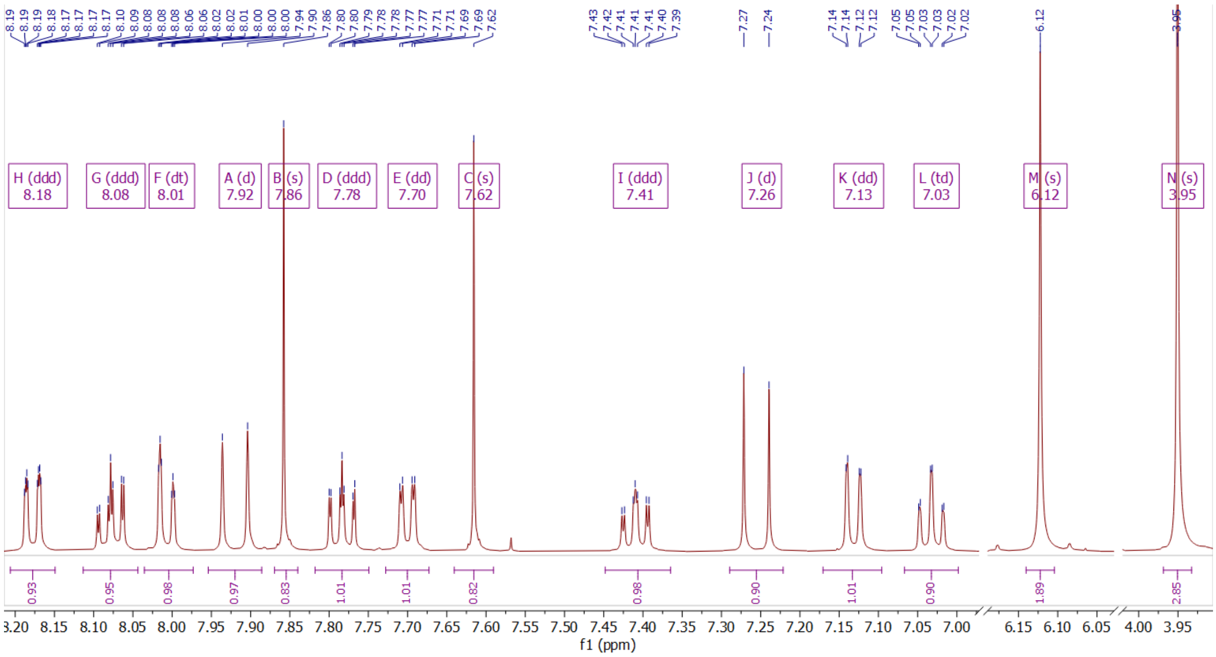

**Figure S3.** $^{1}H$ NMR plot of **BS2**.

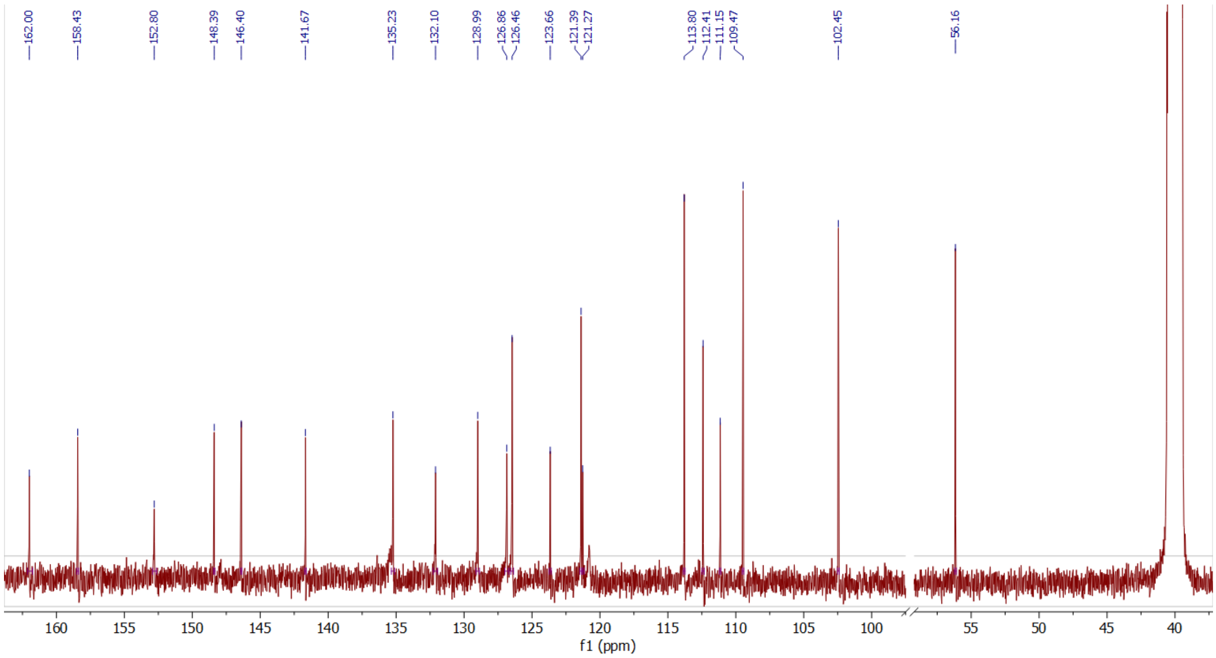

**Figure S4.** $^{13}C$ NMR plot of **BS2**.

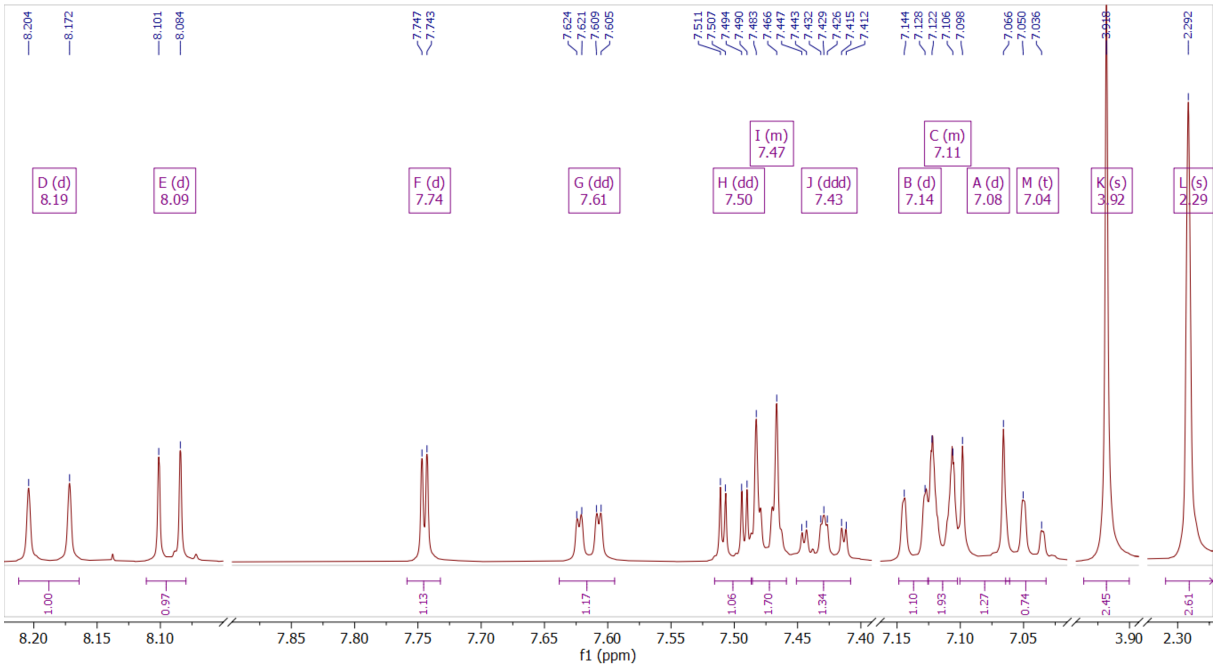

**Figure S5.** $^{1}H$ NMR plot of **BS3**.

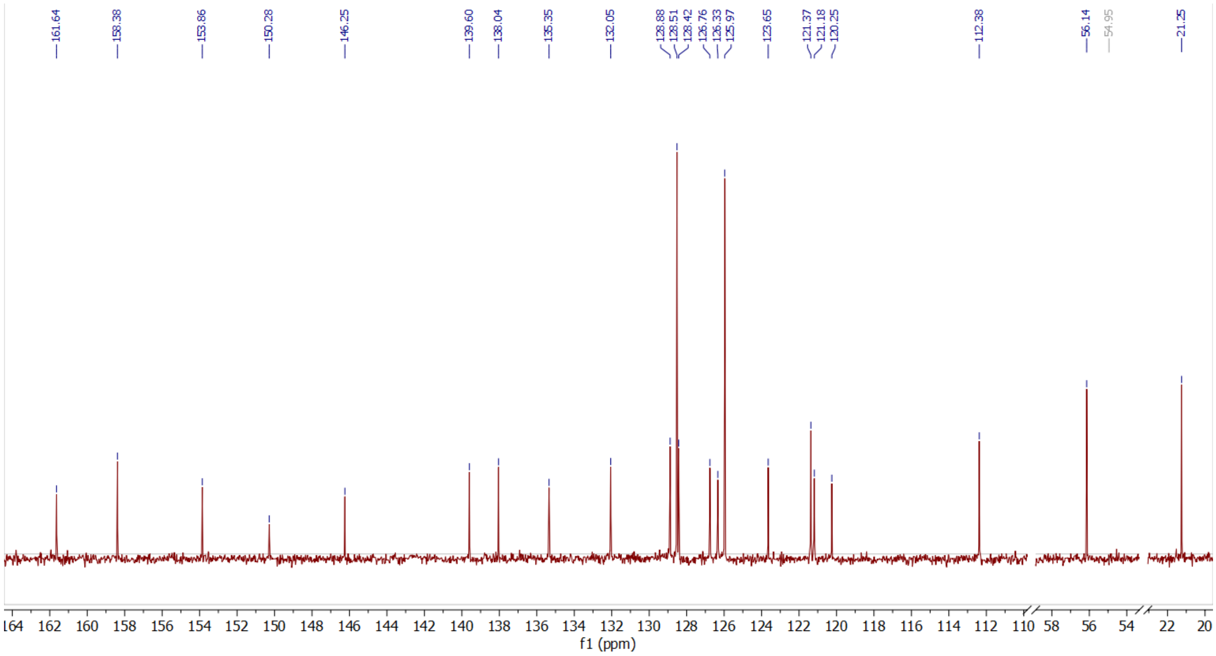

**Figure S6.** $^{13}C$ NMR plot of **BS3**.

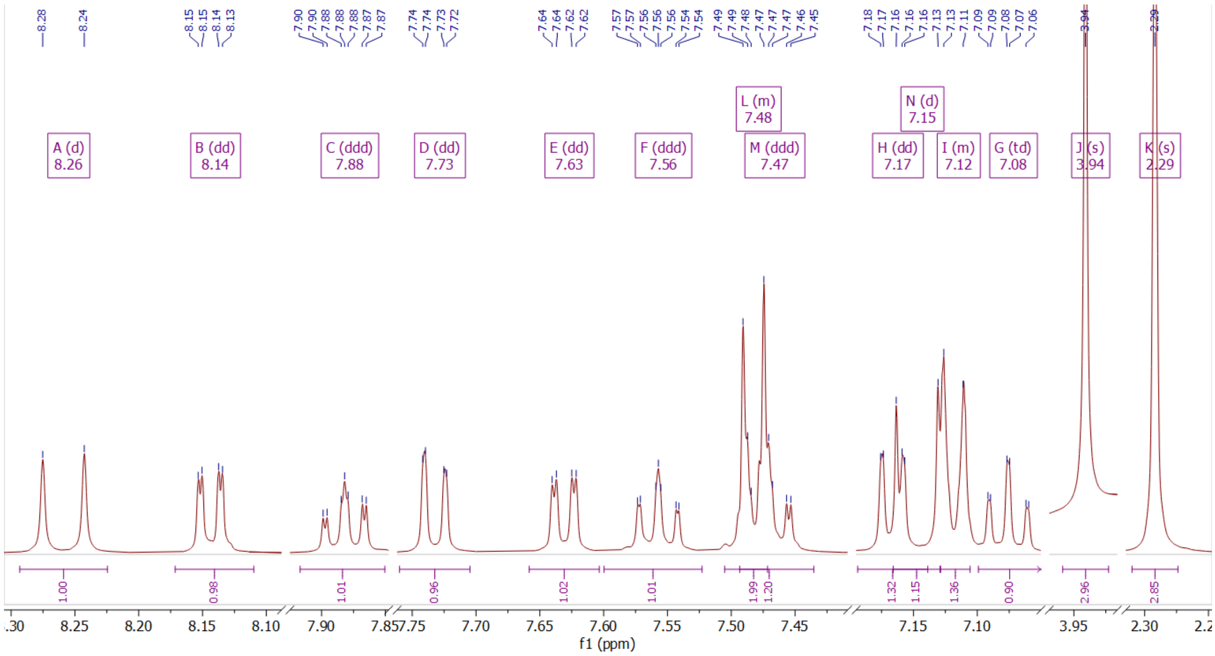


**Figure S7.** $^{1}H$ NMR plot of **BS4.**

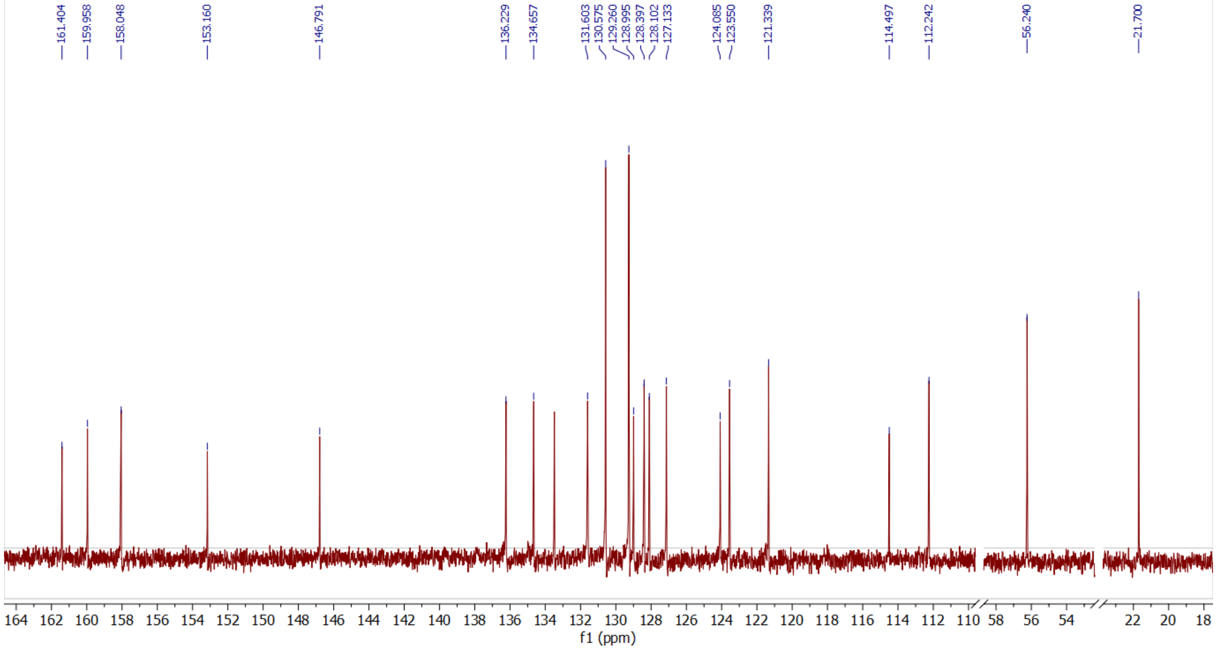


**Figure S8.** $^{13}C$ NMR plot of **BS4**.

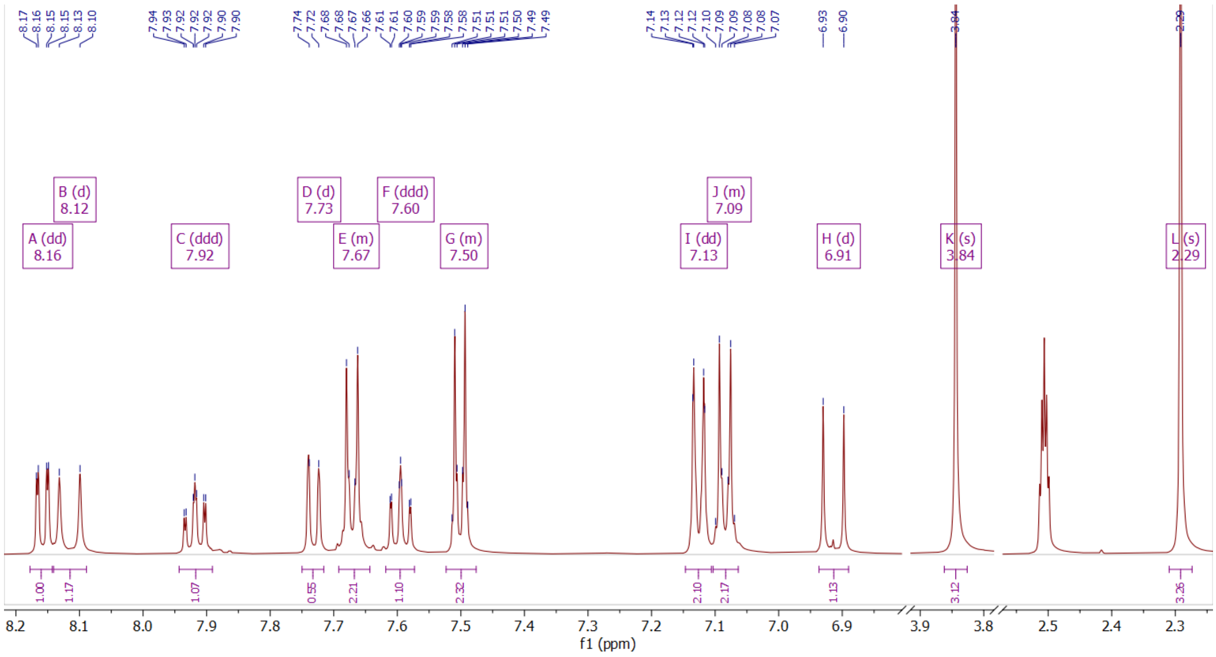

**Figure S9.** $^{1}$H NMR plot of **BS5**.

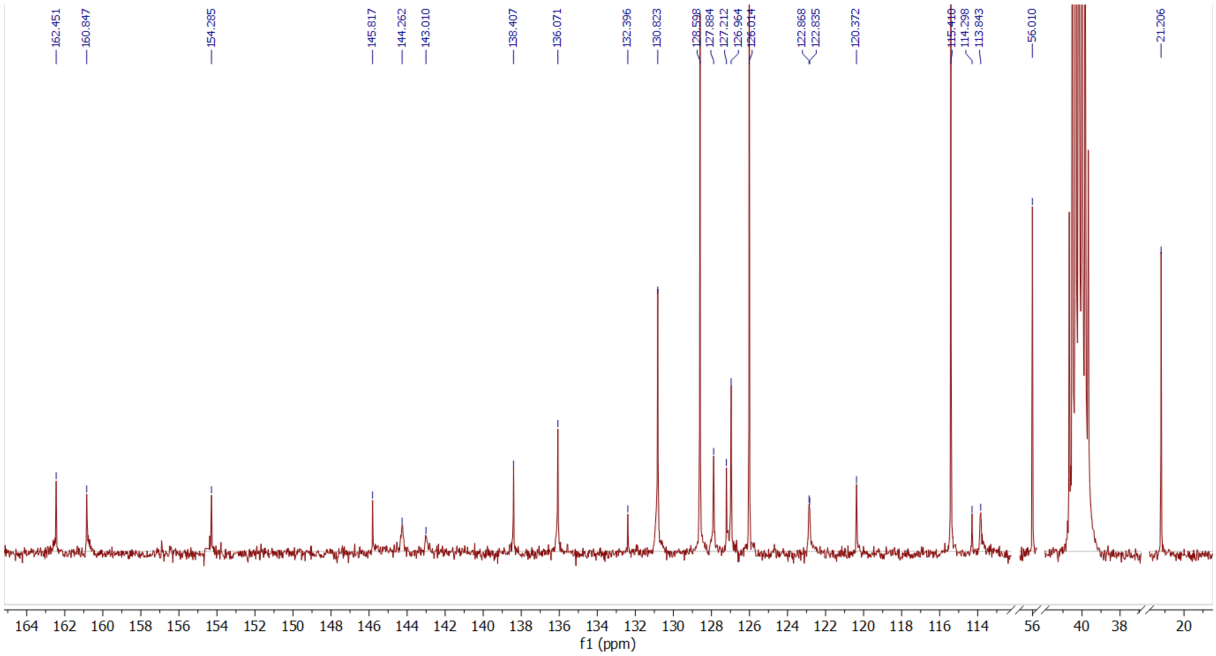

**Figure S10.** $^{13}$C NMR plot of **BS5**.

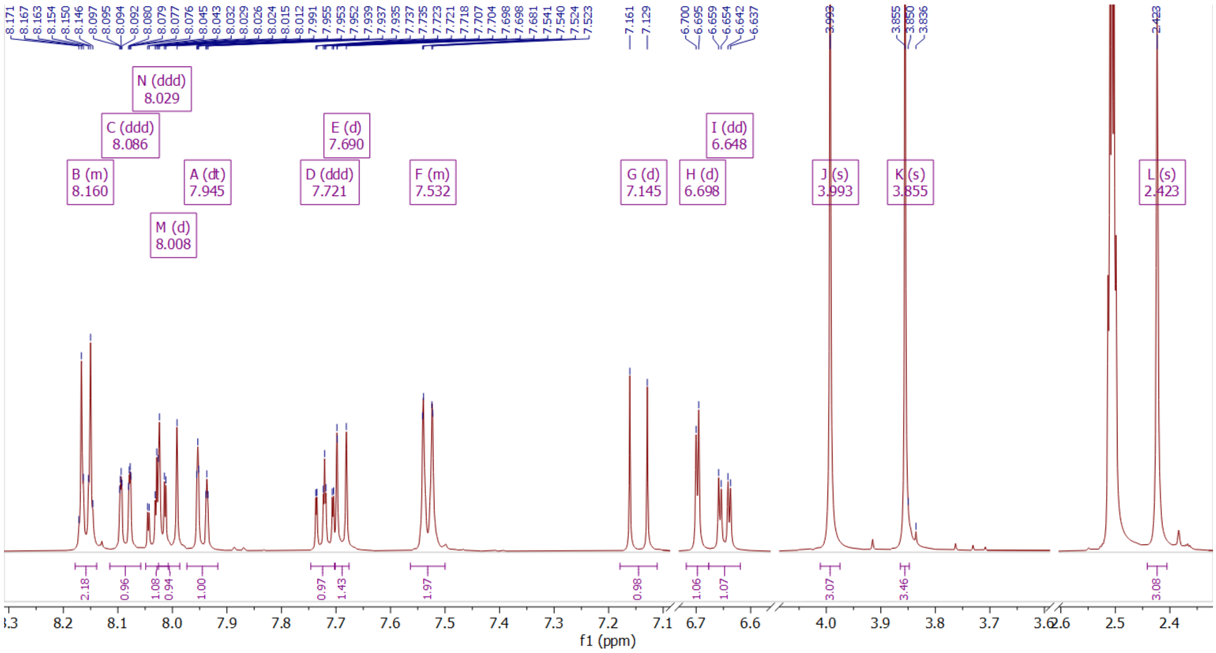

**Figure S11.** $^{1}H$ NMR plot of **BS6**.

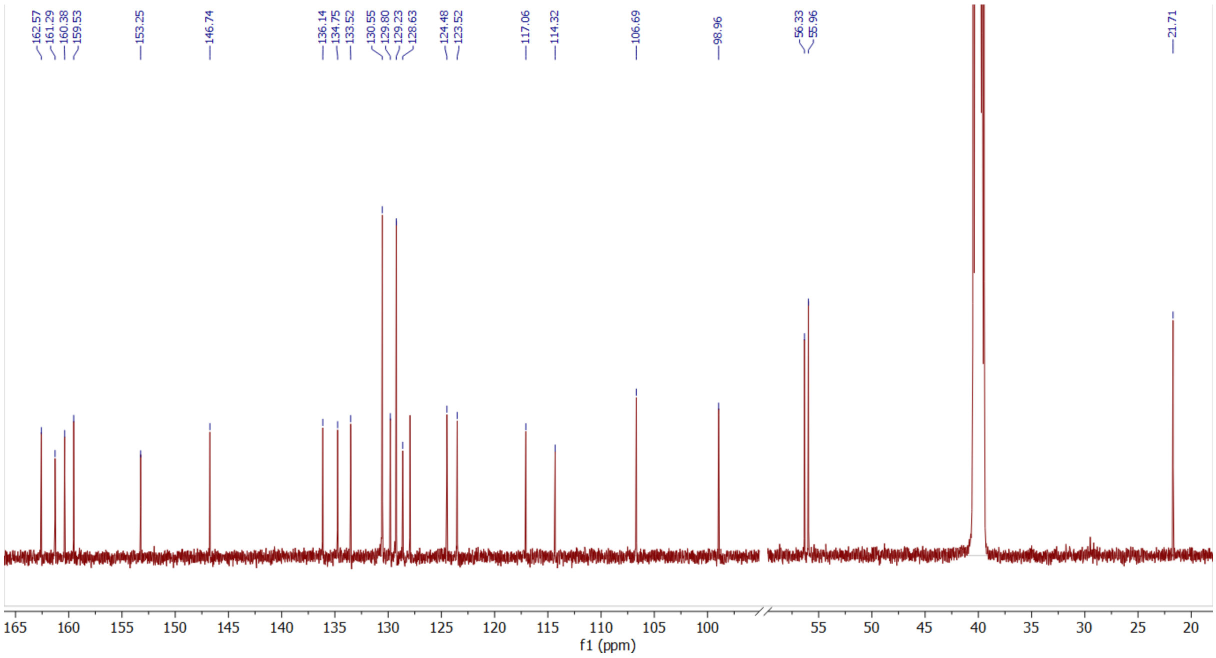

**Figure S12.** $^{13}C$ NMR plot of **BS6**.

**HR-ESI spectra of target compounds:**

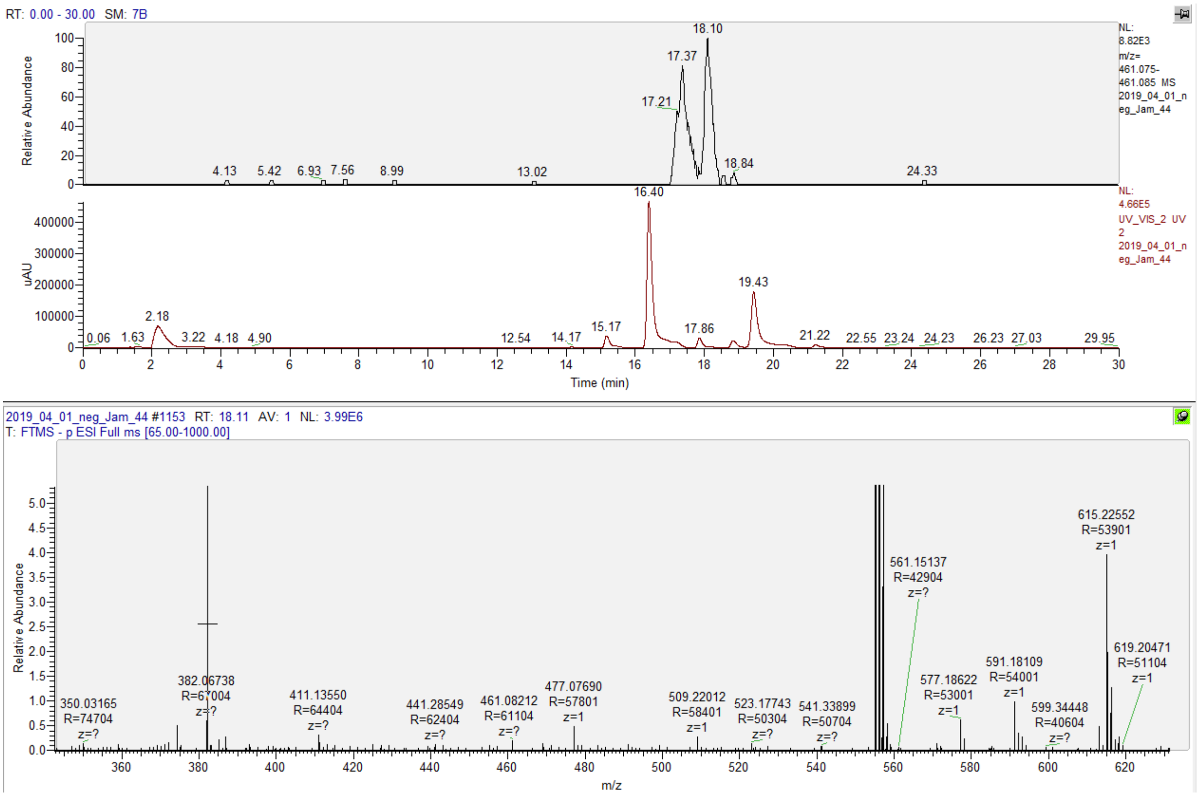

**Figure S13.** HR-ESI spectrum of **BS1**.

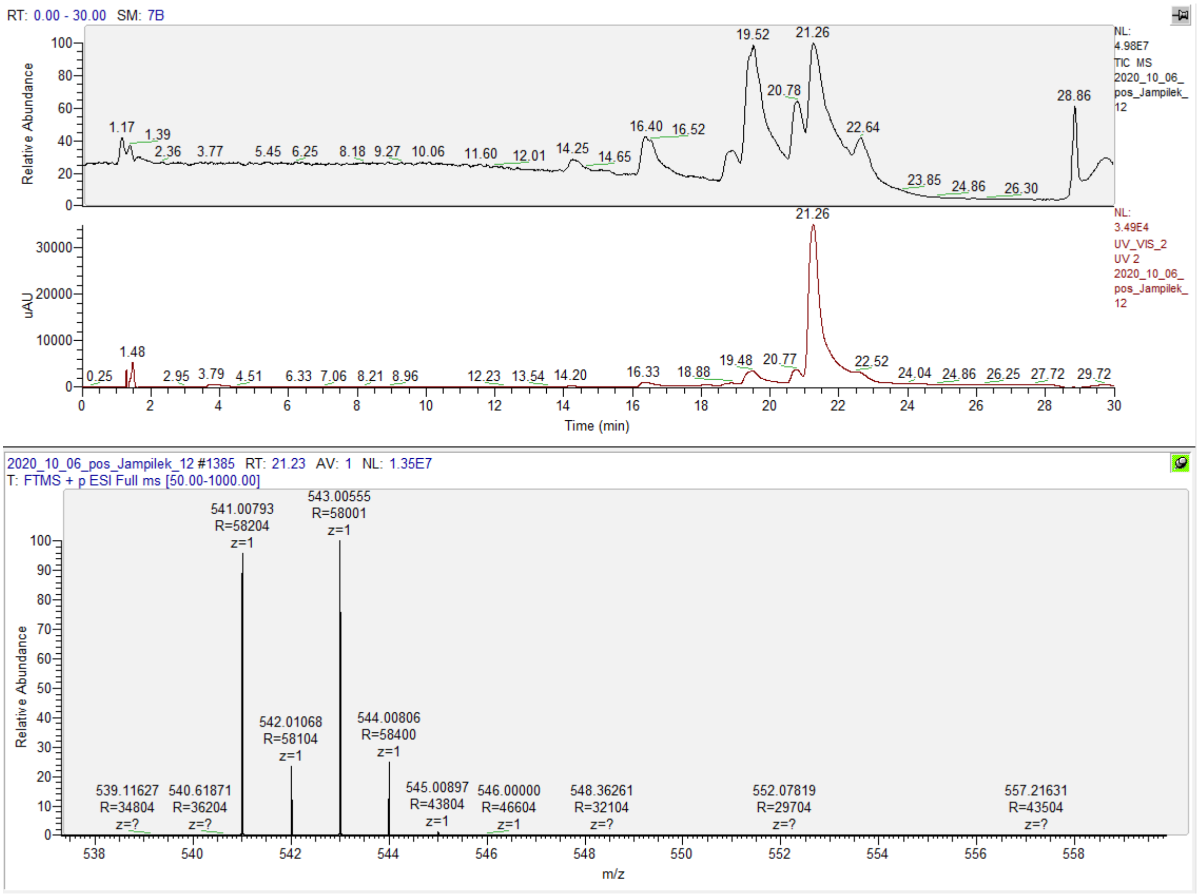

**Figure S14.** HR-ESI spectrum of **BS2**.

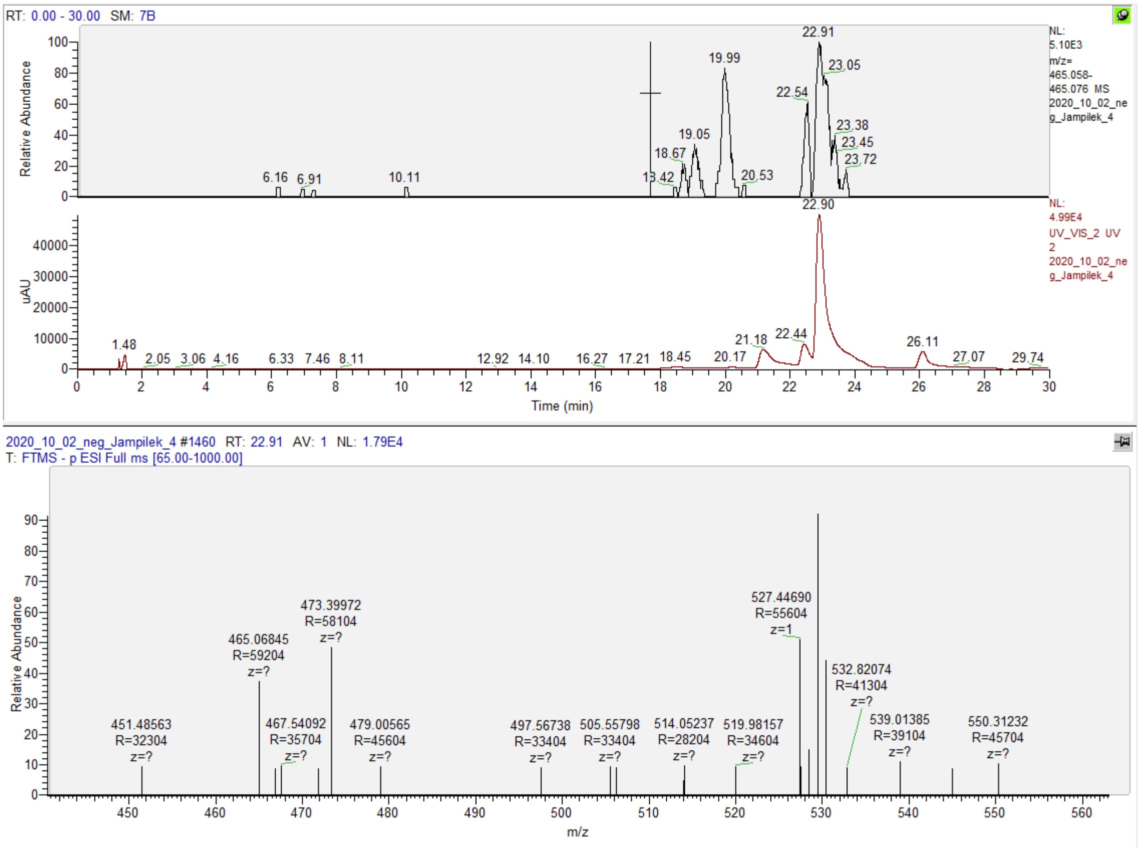


**Figure S15.** HR-ESI spectrum of **BS3**.

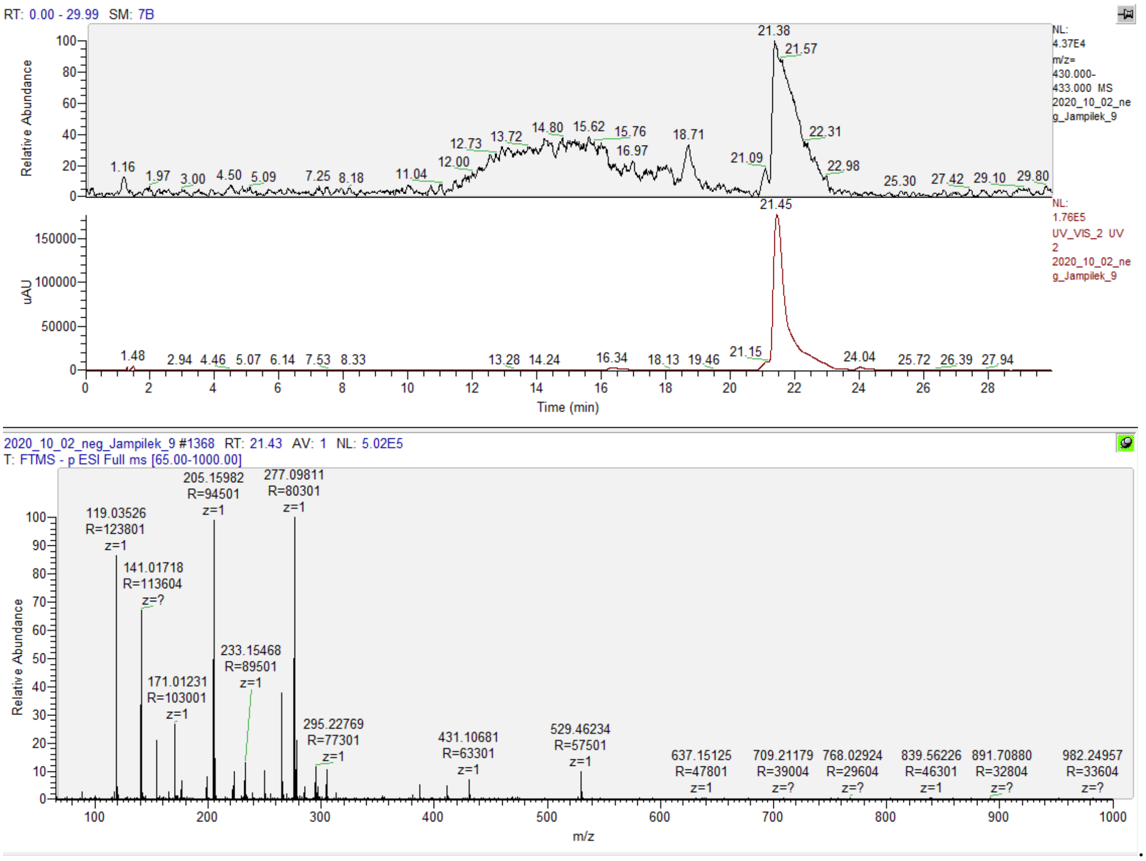


**Figure S16.** HR-ESI spectrum of **BS4**.

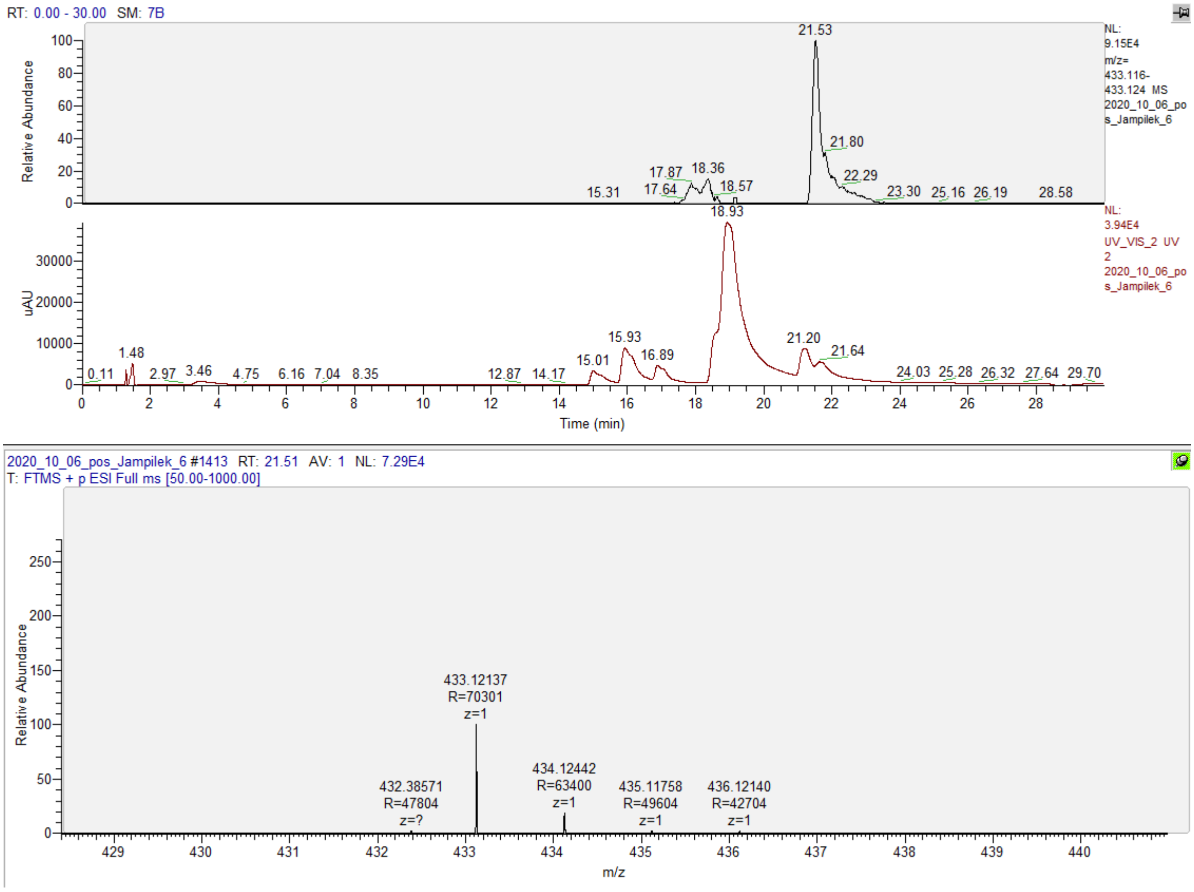


**Figure S17.** HR-ESI spectrum of **BS5**.

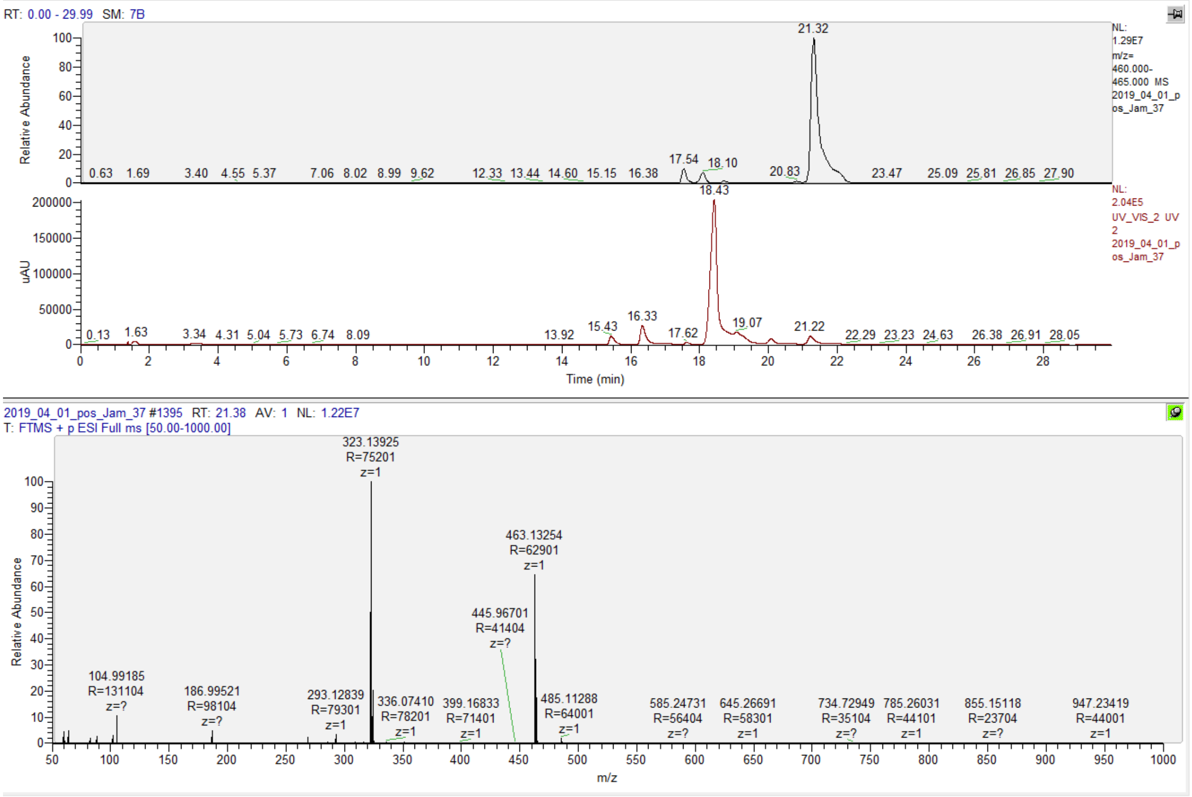


**Figure S18.** HR-ESI spectrum of **BS6**.

## 2. Biological Studies

**Table S1.** Selectivity index of tested derivatives.

| Comp. \ Cell Line | K562 | HCT 116 $p53^{+/+}$ | HCT 116 $p53^{-/-}$ | MCF-7 | A549 | U-251 | PANC-1 |
|---|---|---|---|---|---|---|---|
| **BS1** | 72.91 | 14.25 | 22.27 | 1.51 | 1.00 | 6.61 | 3.15 |
| **BS2** | 63.70 | 13.06 | 11.94 | 0.93 | 1.00 | 6.80 | 5.39 |
| **BS3** | 120.71 | 25.94 | 39.39 | 2.05 | 1.23 | 5.36 | 97.06 |
| **BS4** | 144.51 | 15.95 | 6.71 | 2.74 | 2.20 | 13.11 | 106.38 |
| **BS5** | 2.45 | 1.00 | 1.00 | 1.00 | 1.00 | 1.00 | 1.00 |
| **BS6** | 9.26 | 1.00 | 1.15 | 2.55 | 1.00 | 1.00 | 1.00 |
| **CP-31398** | 3.97 | 0.66 | 0.47 | 0.45 | 0.49 | 0.65 | 0.49 |
| **Imatinib** | 187.97 | 0.56 | 0.49 | 1.00 | 1.00 | 1.00 | 1.00 |

| |
|---|
| **>100** |
| 50–100 |
| 10–50 |
| 1–10 |
| <1 |

**Table S2.** Sequences of primer pairs used in determining the mRNA expression of tested genes.

| Gene | GenBank Accesion no. | Forward Primer (5′→3′) | Reverse Primer (3′→5′) |
|---|---|---|---|
| *IDH1* | NM_001282387 | TCCGTCACTTGGTGTGTAGG | GGCTTGTGAGTGGATGGGTA |
| *GADD45α* | NM_001924.3 | AGTCAGCGCACGATCACTGT | GGATCAGGGTGAAGTGGATCT |
| *calreticulin* | NM_004343.3 | CTGCCGTCTACTTCAAGGA | GAACTTGCCGGAACTGAGAAC |
| *p62* | NM_003900.5 | AGGACGGGGACTTGGTTG | GGCGGGAGATGTGGGTAC |
| *LC3* | NM_022818.5 | CAGCATCCAACCAAAATCCC | CACTGACAATTTCATCCCGAAC |
| *GAPDH* | NM_002046 | GAGTCAACGGATTTGGTCGTA | GCCCCACTTGATTTTGGAG |

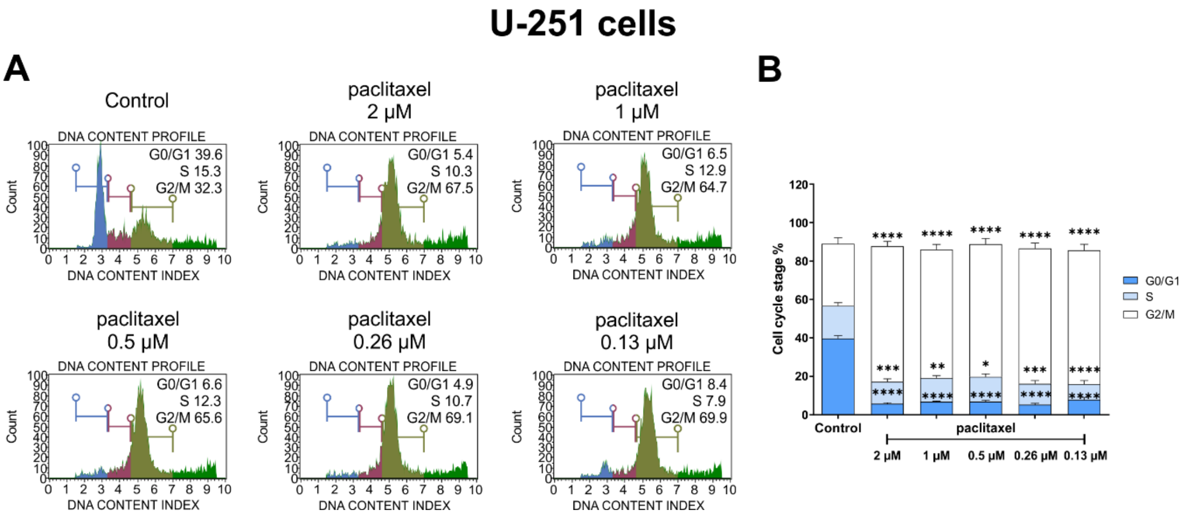


**Figure S19.** Effect of the treatment with the paclitaxel at various concentrations on regulating the cell cycle in U-251 cells. The representative histograms with the distribution of the cells in the respective phases of their cycles for one of several independent experiments (**A**). Data chart with the statistical analysis using a one-way ANOVA with Bonferroni's post-hoc test: * $p < 0.05$, ** $p < 0.01$, *** $p < 0.001$, **** $p < 0.0001$ compared to the untreated cells (control) (**B**).

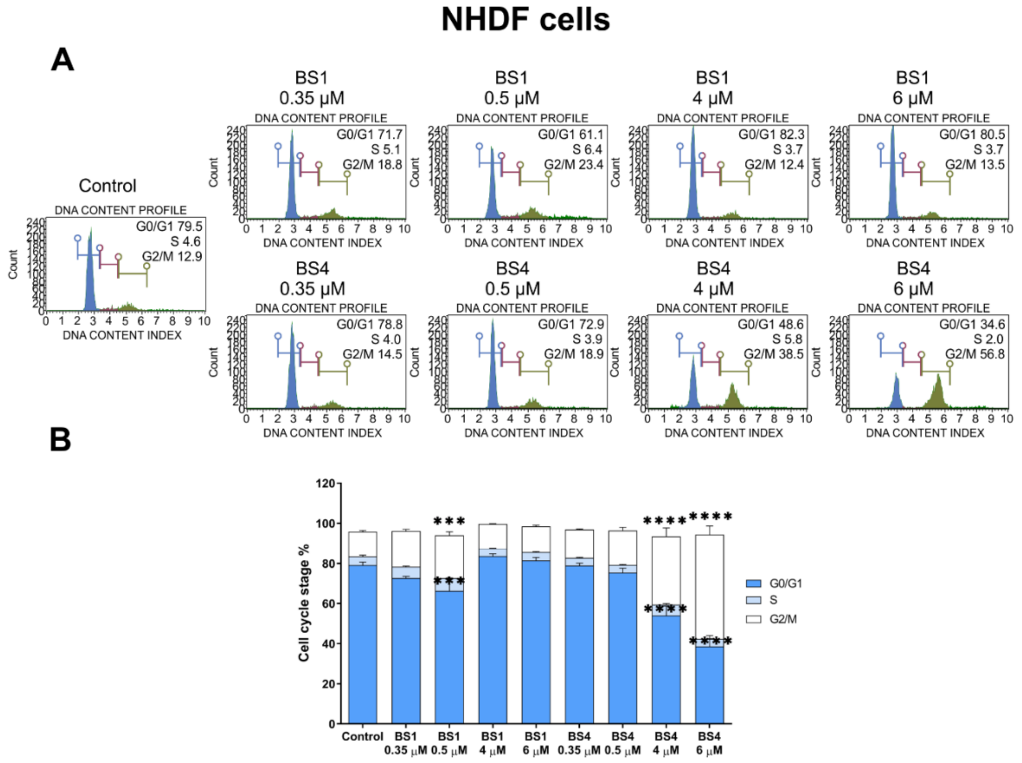


**Figure S20.** Effect of the treatment with the selected compounds (**BS1**, **BS4**) at various concentrations on regulating the cell cycle in NHDF cells. The representative histograms with the distribution of the cells in the respective phases of their cycles for one of several independent experiments (**A**). Data chart with the statistical analysis using a one-way ANOVA with Bonferroni's post-hoc test: *** $p < 0.001$, **** $p < 0.0001$ compared to the untreated cells (control) (**B**).

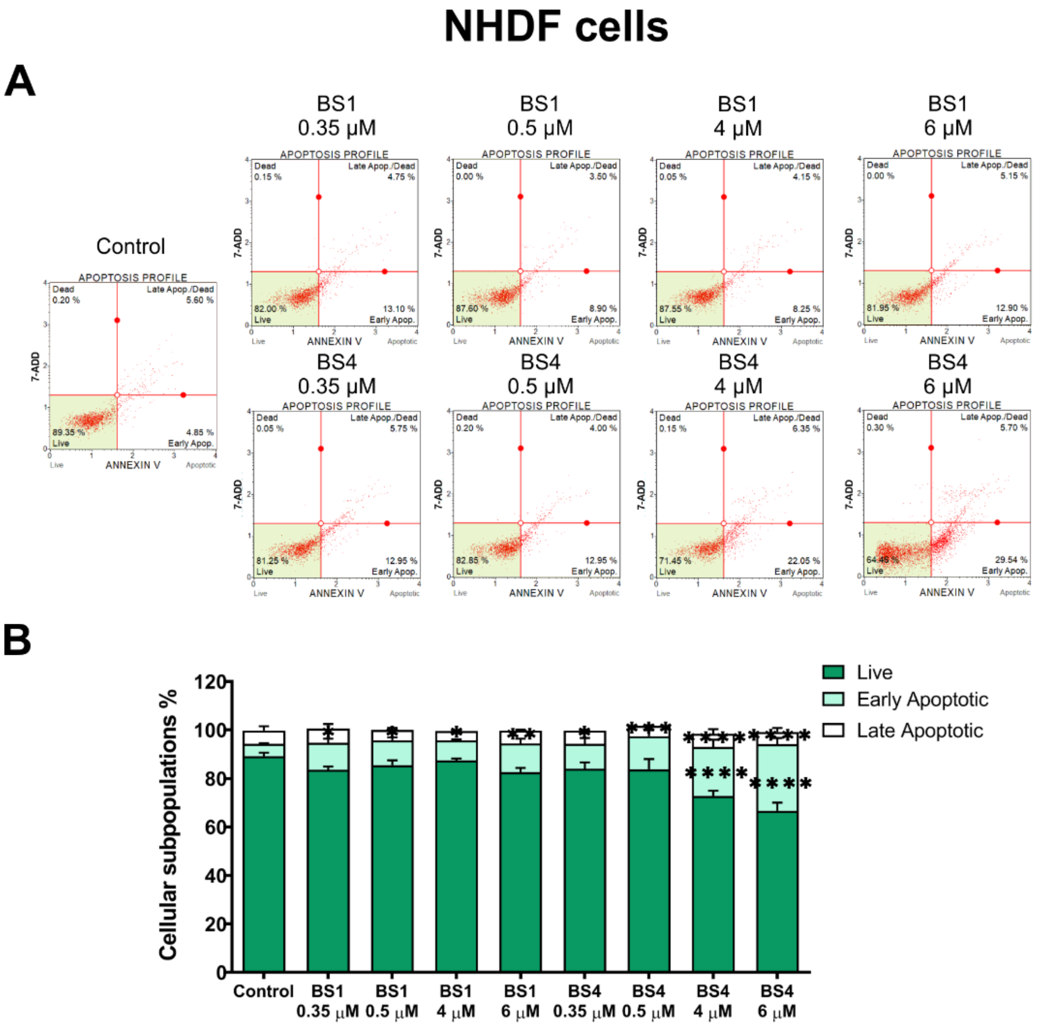


**Figure S21.** Assessment of the effect on the induction of apoptosis in the NHDF cells after a 48-hour incubation with the tested compounds (**BS1**, **BS4**) at various concentrations. The representative histograms from one of several independent experiments include the percentage of live and apoptotic cells (**A**). Data chart with the statistical analysis using a one-way ANOVA with Bonferroni's post-hoc test: * $p < 0.05$, ** $p < 0.01$, *** $p < 0.001$, **** $p < 0.0001$ compared to the untreated cells (control) (**B**).

**The uncropped Western blots and densitometric readings/intensity ratio data:**

Images of the gels prepared during this study. All gels uncropped and unmodified. Proteins relevant to this study are marked in boxes along with reference proteins. Colors used to distinguish appropriate protein pairs.

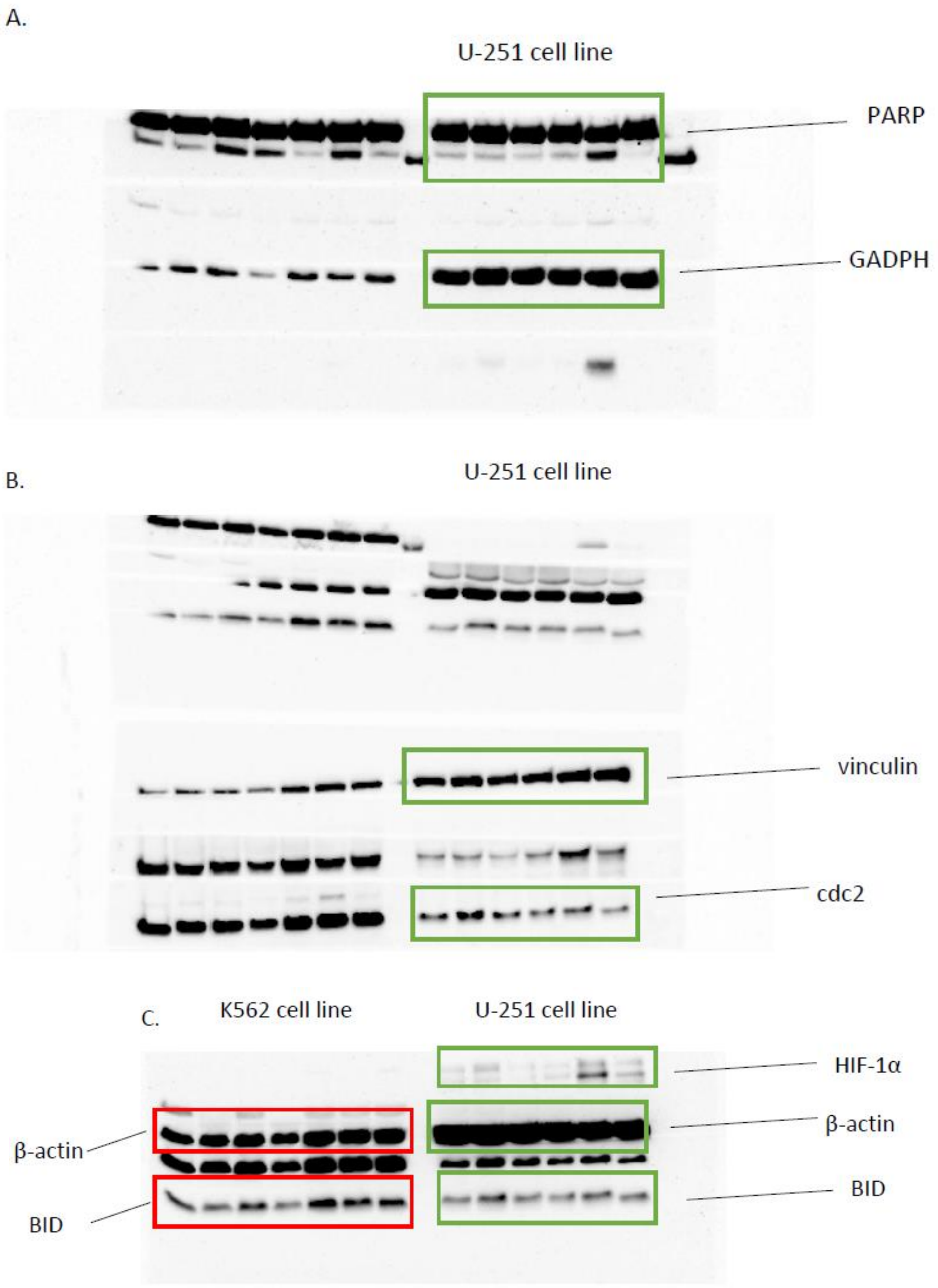

D.

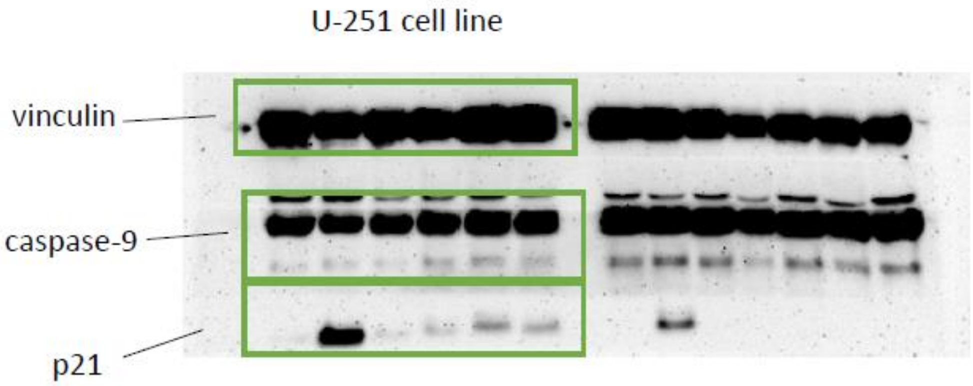
U-251 cell line
vinculin
caspase-9
p21

E.

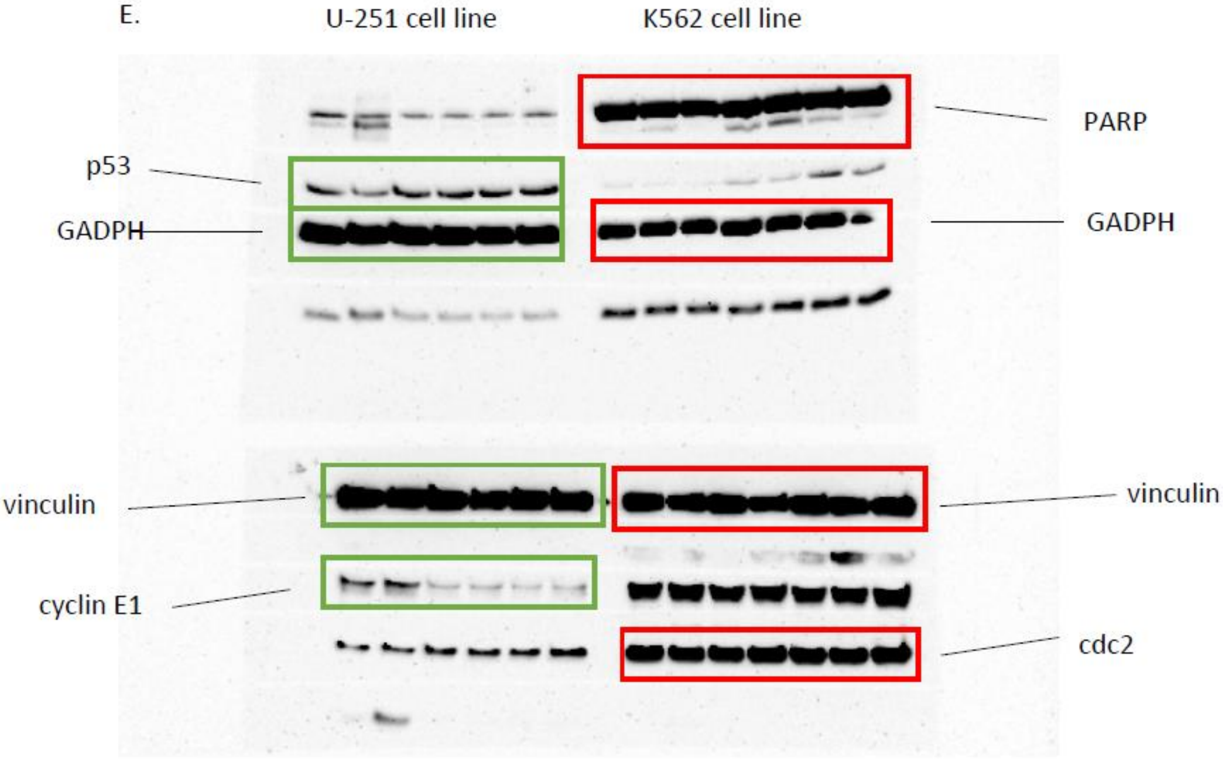
U-251 cell line
K562 cell line
PARP
p53
GADPH
GADPH
vinculin
vinculin
cyclin E1
cdc2

F.

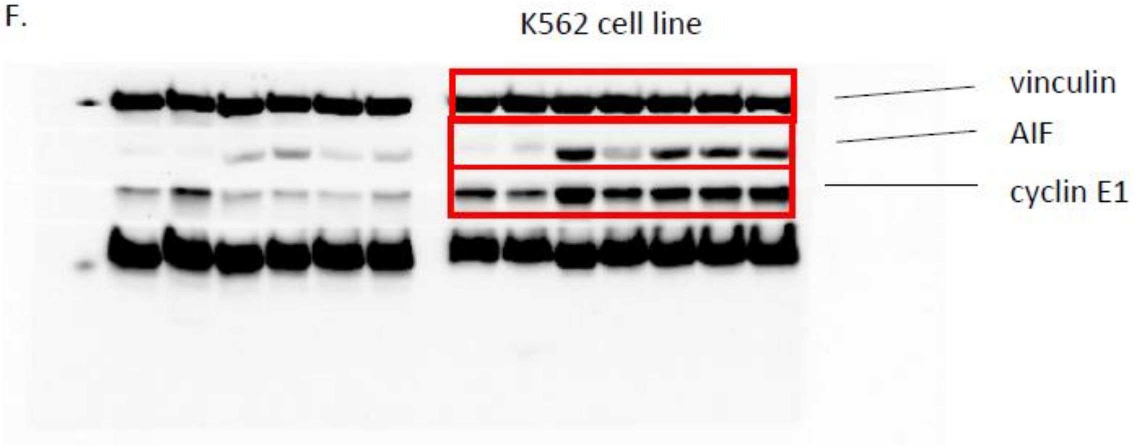
K562 cell line
vinculin
AIF
cyclin E1

G.

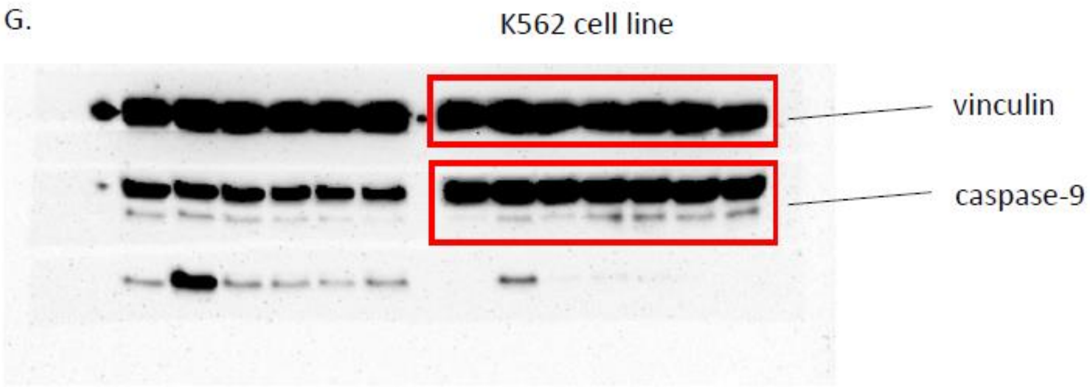


H.

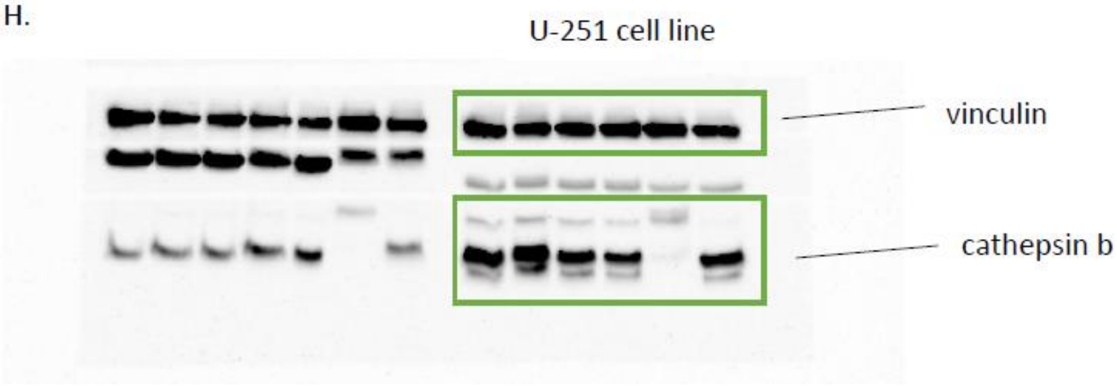


The band intensity of tested proteins (p53, cyclin E1, cdc2, p21, HIF-1α, cathepsin b, PARP, caspase-9, BID, AIF) were analyzed by densitometry readings/intensity ratio, using ImageJ software (NIH) and were normalized to the corresponding reference proteins (GAPDH, vinculin or β-actin) value.

The densitometric readings/intensity ratio data from four/five independent experiments (compared to control):

**U-251 cell line**

| | p53/ Reference | p21/ Reference | cdc2/ Reference | Cyclin E1/ Reference | HIF-1α/ Reference | Cathepsin b/Reference | | |
|---|---|---|---|---|---|---|---|---|
| | 53 kDa | 21 kDa | 34 kDa | 48 kDa | 120 kDa | 44 kDa | 27 kDa | 22 kDa |
| Control | 1.000 | 1.000 | 1.000 | 1.000 | 1.000 | 1.000 | 2.837 | 2.748 |
| BS1 4μM | 1.357 | 1.932 | 1.986 | 0.576 | 0.550 | 2.467 | 2.726 | 2.940 |
| BS2 6μM | 1.448 | 2.783 | 2.282 | 0.392 | 0.661 | 2.641 | 3.172 | 3.717 |
| BS2 4μM | 1.698 | 2.205 | 2.272 | 0.465 | 0.587 | 1.805 | 2.625 | 2.893 |

| | PARP/Reference | | BID/ Reference | Caspase-9/Reference | |
|---|---|---|---|---|---|
| | 116 kDa | 89 kDa | 22 kDa | 47 kDa | 37-35 kDa |
| Control | 1.000 | 0.330 | 1.000 | 1.000 | 0.330 |
| BS1 4μM | 0.871 | 1.044 | 0.675 | 1.167 | 1.485 |
| BS2 6μM | 0.940 | 0.698 | 1.227 | 1.035 | 1.188 |
| BS2 4μM | 0.953 | 0.763 | 0.701 | 1.007 | 1.071 |

**K562 cell line**

| | cdc2/ Reference | cyclin E1/ Reference | AIF/ Reference | BID/ Reference |
|---|---|---|---|---|
| | **34 kDa** | **48 kDa** | **67 kDa** | **22 kDa** |
| Control | 1.000 | 1.000 | 1.000 | 1.000 |
| BS1 0.5μM | 1.122 | 1.110 | 2.737 | 0.695 |
| BS1 0.35μM | 1.234 | 1.273 | 3.809 | 1.049 |
| BS2 0.5μM | 1.206 | 1.270 | 4.160 | 0.762 |
| BS2 0.35μM | 1.338 | 1.640 | 4.141 | 0.965 |

| | PARP/ Reference | | caspase-9/ Reference | |
|---|---|---|---|---|
| | **116 kDa** | **89 kDa** | **47 kDa** | **37-35 kDa** |
| Control | 1.000 | 0.167 | 1.000 | 0.167 |
| BS1 0.5μM | 1.646 | 2.156 | 1.627 | 1.368 |
| BS1 0.35μM | 1.793 | 2.111 | 1.109 | 1.287 |
| BS2 0.5μM | 1.665 | 0.811 | 1.104 | 1.113 |
| BS2 0.35μM | 1.556 | 0.494 | 1.108 | 1.268 |

**Figure S22.** The uncropped Western blots.
